# Climate change has likely already affected global food production

**DOI:** 10.1371/journal.pone.0217148

**Published:** 2019-05-31

**Authors:** Deepak K. Ray, Paul C. West, Michael Clark, James S. Gerber, Alexander V. Prishchepov, Snigdhansu Chatterjee

**Affiliations:** 1 Institute on the Environment (IonE), University of Minnesota, Saint Paul, Minnesota, United States of America; 2 Department of Natural Resources Science and Management, University of Minnesota, Saint Paul, Minnesota, United States of America; 3 now at the Oxford Martin School and Nuffield Department of Population Health, University of Oxford, Oxford, United Kingdom; 4 Department of Geosciences and Natural Resource Management (IGN), University of Copenhagen, Copenhagen, Denmark; 5 School of Statistics, University of Minnesota, Minneapolis, Minnesota, United States of America; Kyungpook National University, REPUBLIC OF KOREA

## Abstract

Crop yields are projected to decrease under future climate conditions, and recent research suggests that yields have already been impacted. However, current impacts on a diversity of crops subnationally and implications for food security remains unclear. Here, we constructed linear regression relationships using weather and reported crop data to assess the potential impact of observed climate change on the yields of the top ten global crops–barley, cassava, maize, oil palm, rapeseed, rice, sorghum, soybean, sugarcane and wheat at ~20,000 political units. We find that the impact of global climate change on yields of different crops from climate trends ranged from -13.4% (oil palm) to 3.5% (soybean). Our results show that impacts are mostly negative in Europe, Southern Africa and Australia but generally positive in Latin America. Impacts in Asia and Northern and Central America are mixed. This has likely led to ~1% average reduction (-3.5 X 10^13^ kcal/year) in consumable food calories in these ten crops. In nearly half of food insecure countries, estimated caloric availability decreased. Our results suggest that climate change has already affected global food production.

## Introduction

Previous assessments of climate change impact on crop yields commonly combine future climate scenarios and process-based crop models to project future yields for a limited number of crops for 2050 or later [1–4]. At higher levels of warming, strong yield losses are predicted in lower latitudes especially for maize and wheat crops [2]. Although these results provide insights into long-term future changes, there are large uncertainties in both the modeled climate projections [5] and in the crop model parameters [6–8]. Hence, the distant time horizon, small number of crops, and coarse resolution limit the results’ utility for stakeholders and policy makers to develop goals and strategies.

Assessing the impacts of recent climate change complements long-term forecasts and identifies which crops and places are already at greater risk. Since the 1970s, global surface temperature warmed at an average of 0.16°C to 0.18°C per decade [9], a rate higher than any period since the industrial revolution. During that same period, we find that the growing season temperature, over all harvested areas for the top ten global crops–barley, cassava, maize, rice, oil palm, rapeseed, sorghum, sugarcane, soybean and wheat–increased 0.5°C to 1.2°C (S1 Table; S1–S4 Figs). Growing season precipitation changes were more variable; from a decrease of 3.4 mm averaged over all sugarcane harvested croplands to an increase of ~19 mm averaged over all oil palm harvested croplands (S1 Table; S1–S4 Figs).

Given the complexities in modeling crop growth response to regional variability in climate and management, previous process based crop modeling efforts have had difficulty reproducing historical crop yields for the few major crops generally studied such as maize, rice, and wheat [7, 10]. Empirical (statistical) global estimates of recent climate change impacts, which some studies have shown to perform equally well as process-based modeling in assessing future impacts on crop yields [11, 12], is available, but only at the coarse national scale [13, 14] or at selected locations, and only for the top few crops [4, 15].

To enable subnational analysis, we first constructed a database of harvested area, yield, weather, and climate statistics building on methods developed previously and accessing publicly available data from individual countries [16]. Crop statistics were compiled from 1974–2013 for ten crops across ~20,000 political units globally (S1 Text). These ten crops account for ~83% of global kilocalorie production from all croplands [17] and represent several major crop group types. (Crops were not distinguished between varieties and managements—see section 1 of S1 Text for more details). Weather and climate statistics (seasonal and annual, normal and extreme, precipitation and temperature) were calculated for each political unit using the CRU TS4.01 gridded global dataset ([18], see S1 Text for further information on how political unit level weather and climate information was constructed). *Historical climate* is defined as the 30-year average weather prior to 1974. *Current climate* is defined as the historical climate plus the addition of the linear trend of the weather for the 35 years ending in 2008, from the year 1974.

We constructed statistical models relating the observed yields to observed weather at each political unit from 1974 to 2008. (The period 2009 to 2013 was set aside for out-of-sample cross validation of model predicted crop yields against observations, at each political unit). We used a time-series analysis (Methods below) in which the influence of technology and management changes were accounted for in linear and quadratic time terms, whereas the normal and extreme temperature and precipitation variations and their interactions were represented with linear and quadratic terms consistent with previous studies [16, 19–21]. The analysis included only those political units that had crop data for each year from 1974 to 2008.

We base quantitative conclusions on models that are statistically significant (ANOVA F-statistic at p < 0.05 level) and are applied within the domain of observed variables used in model construction (Methods below; S5 and S6 Figs). We applied models in the political units in which they were constructed. We confirmed that yield predictions were made only with historical and current climate conditions that were within the range of the observed weather used for model construction (S6 Fig). Further, testing the model residuals showed Gaussian nature of residuals everywhere and their general white noise nature (absence of autocorrelation) (S7 Fig) indicating that these models were robust relative to data quality issues. Cross validation of the model predicted yields, against observations, showed low average (2009 to 2013) harvested-area-weighted errors globally (S2 Table), and generally also low errors at the political units for individual crops (Methods below and S5 Fig). The model coefficients of determination (R^2^) at the global level ranged from 0.76 (sorghum) to 0.87 (rice) (S3 Table; S8 Fig).

The potential impact of climate change at each political unit is the difference in crop yield under current, and under historical climate conditions. This yield change information at the political unit was then converted to production changes using the average harvested area information over the 2003 to 2008 period at each political unit. Then all the political unit level climate-driven production changes in a country were summarized to give the country-level climate-driven production changes. Finally, we translated the country-level production changes to country-level consumable food calorie changes. In this process we accounted for harvested calories that returned as food calories after getting processed through animals as feed, through food processing, and calories directly consumed with little to no processing (S1 Text).

## Results

### Global scale patterns

**Fig 1:**
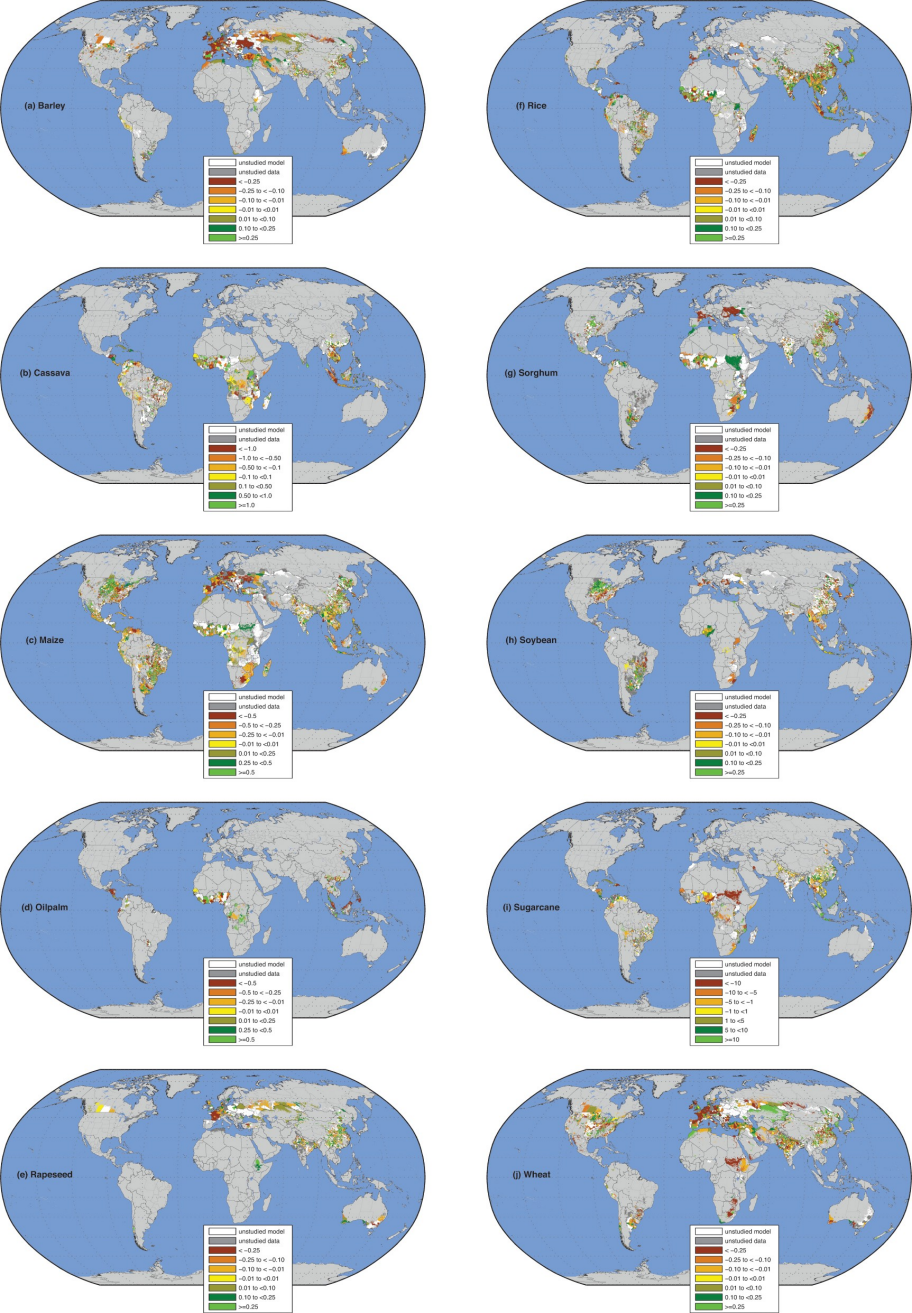
Impact of mean climate change on crop yield (tons/ha/year). Brown colors denoted reduction in yield and green colors indicate gains in yield due to mean climate change. (a) barley; (b) cassava; (c) maize; (d) oil palm; (e) rapeseed; (f) rice; (g) sorghum; (h) soybean; (i) sugarcane; and (j) wheat. White areas are where the study was not conducted due to model (unstudied model) and dark gray areas are where the study was not conducted because of data (unstudied data). Light gray areas are where we do not have any report of the crop being harvested or the crop is insignificant and is mapped as background color in land areas. Oceans, seas, large lakes, and large water bodies are mapped in blue color.

Although recent changes in mean climate occurred across all croplands (S1 Table; S1–S4 Figs), the statistical relationship between weather and crop yields was significant in 54%-88% of harvested areas globally across crops (p < 0.05, Table 1, Fig 1; for model performance see S1–S4 Tables and S7 and S8 Figs). We restricted analysis of mean climate change to these harvested areas (Table 1, Fig 1). There are differences in the spatial extent of the statistically significant harvested croplands among crops and regions. For example, in 88% (125 million hectares (Mha)) of rice-harvested croplands the relationship was significant but only in 54% (22 Mha) of sorghum harvested areas globally. Within North and Central America recent climate change impact was consequently significant over 89% of maize but 71% of wheat-harvested areas (Table 1).

**Table 1:** Impact of mean climate change summarized by large regions.

	North and Central America	Caribbean and South America	Western and Southern Europe	Eastern and Northern Europe	Northern Africa	Sub-Saharan Africa	Central and Eastern Asia	Western, Southern and South-eastern Asia	Oceania	Global
Percentage of harvested areas where model is significant at p < 0.05										
Barley	61	63	97	65	95	14	90	92	40	**74**
Cassava	96	52	NA	NA	100	62	34	90	NA	**66**
Maize	89	85	94	58	100	57	75	77	55	**77**
Oilpalm	90	66	NA	NA	NA	55	79	84	NA	**72**
Rapeseed	53	100	96	34	NA	100	75	75	66	**66**
Rice	92	87	93	18	100	77	89	88	100	**88**
Sorghum	59	70	100	74	100	39	85	38	100	**54**
Soybean	87	92	94	22	100	88	56	46	60	**81**
Sugarcane	43	79	74	NA	94	63	76	69	6	**70**
Wheat	71	63	90	43	100	89	88	90	68	**75**
Yield change (kg/ha/year averaged over significant model areas)										
Barley	-131	124	-726	-355	-66	-64	38	-17	-94	**-269**
Cassava	-270	130	NA	NA	301	275	575	-1070	NA	**-83**
Maize	48	129	-614	-1839	-264	-148	396	38	-154	**2**
Oilpalm	-1188	-130	NA	NA	NA	-2	-66	-3930	NA	**-2551**
Rapeseed	-13	256	-400	188	NA	264	143	27	10	**14**
Rice	-9	-34	-233	-98	-131	-75	66	-31	335	**-13**
Sorghum	276	1	-1010	-225	136	17	324	20	-856	**53**
Soybean	108	151	-709	-209	352	-20	7	-72	-240	**104**
Sugarcane	2986	2251	3065	NA	-6085	-2912	4628	-529	5538	**982**
Wheat	-48	-66	-537	-145	278	-48	175	-25	-125	**-33**
	Production change (million tons (MT)/year)									
Barley	-0.41	0.05	-4.85	-4.58	-0.23	-0.01	0.09	-0.13	-0.13	**-10.20**
Cassava	-0.04	0.15	NA	NA	0.00	1.90	0.05	-2.98	NA	**-0.92**
Maize	1.73	1.72	-2.35	-6.14	-0.29	-2.03	7.00	0.53	-0.01	**0.17**
Oilpalm	-0.11	-0.03	NA	NA	NA	-0.01	0.00	-19.64	NA	**-19.79**
Rapeseed	-0.04	0.00	-0.76	0.20	NA	0.01	0.68	0.11	0.01	**0.21**
Rice	-0.01	-0.14	-0.08	0.00	-0.09	-0.44	1.78	-2.66	0.02	**-1.62**
Sorghum	0.70	0.00	-0.09	-0.01	0.92	0.12	0.10	0.07	-0.65	**1.16**
Soybean	2.58	4.00	-0.12	-0.06	0.00	-0.02	0.04	-0.20	0.00	**6.22**
Sugarcane	1.59	10.84	0.00	NA	-1.25	-1.59	4.38	-2.51	0.13	**11.60**
Wheat	-1.01	-0.29	-6.17	-2.56	1.93	-0.10	5.28	-1.20	-0.83	**-4.95**
Percentage yield / production changed w.r.t current average (at fixed all current harvested areas)										
Barley	-2.5	4.0	-16.1	-9.1	-6.8	-0.6	1.6	-0.9	-2.3	**-7.9**
Cassava	-2.9	0.5	NA	NA	18.0	1.7	1.2	-5.6	NA	**-0.5**
Maize	0.5	2.7	-6.3	-24.5	-4.3	-5.8	5.1	1.0	-1.2	**0.0**
Oilpalm	-7.2	-0.6	NA	NA	NA	0.0	-0.4	-15.9	NA	**-13.4**
Rapeseed	-0.4	6.8	-11.4	3.1	NA	24.9	5.9	1.9	0.6	**0.5**
Rice	-0.1	-0.7	-3.2	-0.4	-1.3	-3.1	0.9	-0.8	4.1	**-0.3**
Sorghum	4.3	0.0	-18.2	-9.5	17.9	0.7	4.9	0.9	-30.5	**2.1**
Soybean	3.3	5.4	-21.2	-3.8	10.9	-1.6	0.2	-3.2	-6.3	**3.5**
Sugarcane	1.7	2.5	2.7	NA	-5.1	-3.9	5.3	-0.6	0.4	**1.0**
Wheat	-1.3	-1.6	-8.7	-2.1	12.0	-2.3	4.5	-0.9	-5.8	**-0.9**
Percentage kilocalories changed w.r.t current kilocalories consumed from the crop (only countries reporting consumption as per the FAO Food Balance Sheets are included & trade is not accounted). Also see S4 Table										
Barley	-12.9	1.5	-218.0	-67.9	-9.8	-0.3	0.9	-3.3	-952.7	**-14.9**
Cassava	-14.5	0.7	NA	NA	17.7	3.1	0.4	-5.7	NA	**0.5**
Maize	2.3	1.5	-10.6	-50.2	-2.0	-4.0	6.0	1.0	-1.9	**-0.7**
Oilpalm	-6.3	-1.0	NA	NA	NA	-0.6	0.0	-219.0	NA	**-56.6**
Rapeseed	-2.6	3.8	-34.8	8.0	NA	34.5	7.3	1.7	1.6	**1.1**
Rice	-0.8	-0.9	-6.1	-0.3	-2.5	-2.6	1.4	-1.2	5.5	**-0.4**
Sorghum	25.5	-0.8	0.0	0.0	20.0	0.5	4.8	0.8	0.0	**3.6**
Soybean	6.0	21.0	-3.1	-4.9	0.5	0.6	0.1	-1.0	-1.3	**4.7**
Sugarcane	0.7	4.0	0.0	NA	-3.3	-2.1	5.4	-0.6	1.6	**0.7**
Wheat	-2.0	-1.3	-8.4	-2.8	6.1	-0.8	2.2	-0.8	-11.8	**-0.5**

Averaged globally, yields changed between -2551 (oil palm) to +982 (sugarcane) kg/ha/year (Table 1). The percentage change in recent yield over all harvested croplands ranged from -13.4% (oil palm) to +3.5% (soybean). Among the top three global cereals, recent yields have decreased for rice (-0.3% or ~-1.6 million tons (MT) annually) and wheat (-0.9% or ~-5.0 MT annually) and increased negligibly for maize (0% or ~0.2 MT annually). This translates to an annual 0.4%, 0.5% and 0.7% decrease in consumable food calories available from rice, wheat and maize respectively globally.

Recent climate change generally decreased yields across Europe, Sub-Saharan Africa and Australia, increased yields in Latin America, and had mixed responses in North and Central America and in Asia (Fig 1).

### Europe

Yields for all the dominant (non-tropical) crops in western and southern Europe decreased 6.3–21.2% because of climate change (Table 1, Fig 1). This may partially explain the stagnation of yields in Europe [22]. We observed a decrease in the yields of major crops–wheat, barley, maize and rapeseed–for parts of the steppe region in European Russia and in the grain belt of Western Siberia agreeing with case studies [23]; annual temperature in the Russian Federation has increased since the 1970s at ~0.4°C/decade [23] (S2 Fig). Barley, maize and sorghum productivity has been negatively affected by climate change in Ukraine, confirming observations [24]. Annual yield losses in western and southern Europe are high though exceptions abound as in Andalucía in southern Spain where wheat yields gained from mean climate changes (Fig 1). Similarly, in eastern and northern Europe yield losses are widespread for maize (-24.5%), barley (-9.1%), and wheat (-2.1%). The large yield / production losses across crops in France reduced consumable food calories production in these ten crops by ~24% or ~-7% of all food calories consumed (S4 Table). Large reductions in consumable food calories in these ten crops due to climate change have also occurred in Germany (~-11%), Spain (~-4%), Italy (~-7%) as well as in other major Western European agricultural countries (S4 Table). In Eastern and Northern Europe the largest reductions in consumable food calorie from these ten crops due to mean climate changes occurred in Hungary (~-35% or ~-10% overall), Romania (~-18% or ~-7% overall), and Ireland (~-12% in these ten crops or ~-3% overall).

### Sub-Saharan Africa

Of the major sub-Saharan African crops, maize provides the largest percentage of food calories followed by sorghum, cassava and sugarcane. Maize and sugarcane yields decreased by 5.8% and 3.9%, respectively. In contrast, recent climate change caused yields to increase in the more heat- and drought-tolerant sorghum (0.7%) and cassava (1.7%). Maize yield losses are highest in South Africa (-22%), with the highest losses occurring in the provinces of The Free State and North West (Fig 1). Overall in Sub-Saharan Africa maize yields have decreased but cassava yields increased in response to climate changes, though not everywhere. For example cassava yields decreased in the central to southern parts of Madagascar but increased in northeastern Madagascar. Though Eastern Africa in general had reductions in cassava yields, in Tanzania this was true only in its eastern districts and in the western districts cassava yields benefitted from mean climate changes. This apparent heterogeneity in yield response is seen also in Western Africa. For example in the southern districts of Togo maize yields decreased but in the northern districts maize yields benefitted from mean climate change. Consumable food calorie production from these ten crops was reduced nearly 12% (or ~-8% across all food calories) in South Africa. Large decreases in consumable food calories across all ten crops also occurred in Ghana (~-8%) in western Africa, in Zimbabwe (~-10%) in southern Africa, but increased in Tanzania (~2%) in eastern Africa (S4 Table). In some cases, as in Ghana, gains in consumable calories in maize and rice due to climate change was wiped off from losses in cassava consumable calories leading to overall decreases in consumable food calories. Overall in entire sub-Saharan Africa ~1.4% reduction in food calories in these ten crops or ~0.8% reduction across all consumed food calories from these ten crops occurs on average annually due to climate change.

### Oceania

In the Oceania region we estimated a ~9% reduction in current Australian wheat yields (broadly agreeing with *Hochman et al*. *2017* [25]; S4 Table) as well as reductions in barley, maize, sorghum and soybean yields, but overall increases in rapeseed, rice, and sugarcane yields (Fig 1). Overall, climate change reduced Australian consumable crop calorie production in the ten crops by ~6% (or ~-3% in overall calories) annually.

### North, Central and South America

In North and South America the broad pattern shows benefits to crop yields from climate change especially in commercial crops such as maize, oil palm, soybean and sugarcane (Table 1). Some sub-regions here tend to stand out, for example the consistent yield losses across crops in eastern and southern United States agreeing with previous studies [26, 27] and in the northern parts of the South American continent. Overall in the United States barley, rice and wheat yields reduced whereas maize, sorghum, soybean and sugarcane yields increased. In some countries such as in Canada, Panama, Honduras and Belize consumable food calories decreased whereas in Guatemala the change was insignificant (~0%). Recent climate change increased total consumable calories in the United States and Mexico. Major losses in consumable food calories have occurred in the Dominican Republic, Ecuador, Bolivia, Uruguay and Venezuela whereas in Brazil, Argentina Paraguay and Cuba consumable food calories overall increased.

### Asia

Climate change’s impacts on crop yield and consumable calories in Asia are varied. In China, mean climate changes overall benefitted crop yields and increased consumable food calories in these ten crops by ~2% (or ~1% across all consumable food calories), though there are exceptions such as decreases in rice yields in Guangxi and Fujian or wheat yields in Sichuan and Guizhou (Fig 1). Consistent with *Tao et al*. *2017* [28] and *Meng et al*. *2014* [29] we find that wheat yields in large extent of the Huang-Huai-Hai plains and maize yields in Heilongjiang province similarly benefitted from climate change. In India we found some states with a persistent pattern of yield losses across all major crops as in the core Green Revolution state of Haryana and western Uttar Pradesh, and for rice in southern India (Tamil Nadu and Kerala states) with overall production losses in India in wheat (-0.7% or ~-0.5 MT) and rice in India (-2.1% or ~-2.2 MT broadly agreeing with [30]). Consumable food calories reduced in India ~1.2% in these ten crops and ~0.8% overall on average annually. Losses in rice production have also occurred in Vietnam (~-1.0 MT) and in the Laguna province of Philippines (~11kg /ha/year or -0.2% in yields; Fig 1f), broadly agreeing with reported station experiments [31], although overall rice production increased in the Philippines (S4 Table). Wheat production also decreased in Turkey (~-0.8 MT). Climate change has reduced consumable food calories in numerous Asian countries both food secure (such as in Iran and Israel) and insecure (as in Bangladesh, Nepal and India).

The impact on crop yields on account of only temperature change (holding the precipitation variables at historical climatological values) is mapped in S9 Fig, whereas the impact due to only precipitation change (holding the temperature variables at historical climatological values) is mapped in S10 Fig. Temperature only effects are stronger in some areas such as Europe and East Asia whereas precipitation only effects are equally strong as in sub-Saharan Africa, South Asia and Australia; these are not additive results and provide only an indication of the relative importance of temperature versus precipitation changes (S4 Table).

### Impact on food security

Specifically when assessing the impact of recent climate change on crop production in countries where hunger is prevalent [32] we found the following: across the 53 countries with a hunger index of serious, alarming, or seriously alarming [32] in 2008, we find that recent climate change had decreased consumable calories in 27 countries and increased in 26 (S4 Table). Losses in consumable calories compared to total consumed food calories annually were particularly great in southern parts of the African continent, such as in Zimbabwe (-7.2%), Malawi (-6.5%), and Mozambique (-2.8%); in western Africa such as in Mali (-3.9%) and Ghana (-3.8%); and in Asia such as in India (-0.8%), and Nepal (-2.2%). Globally the average annual change is large for those consuming these ten crops (~ -1%) but not negligible across all consumable food calories as well (~ -0.5%). Although this metric does not address food access, nutrition, and other components of food security [33], it suggests that climate change has increased the risk of food insecurity in many food insecure countries.

## Discussion and conclusions

The Intergovernmental Panel for Climate Change—Assessment Report (AR) 5 [2] (IPCC AR5) on climate change impact on crop yield/production notes that between the AR4 and AR5 the required connection of climate change impact to food security impact was missing. Our study directly addresses this by translating the potential impact of recent climate change on crop yields (Fig 1) to consumable food calories change in each country (Table 1, S4 Table). While translating crop production change to consumable food caloric change, we accounted for the current dietary consumption pattern of individual crops per country, including bookkeeping for directly and indirectly consumed calories in each country [34]. We found that out of the studied 53 countries with a hunger index of serious, alarming, or seriously alarming [32] in 2008, 27 countries (or ~51%) had decreased consumable calories due to mean climate changes (~ -0.4% in these ten crops or ~ -0.3% across all food calories consumed across all 53 countries studied–S4 Table). Though we detected subnational level crop yield and production changes (Fig 1), determining consumable food caloric changes at that level would require data not available globally: subnational dietary patterns, evaluation of the climate change impact in the entire food supply chain [35] and socio-economic conditions [36]. These individual issues should be explored in future studies to understand mean climate change impacts on local scale food security.

Linear and extreme trends in weather were captured using linear and quadratic terms whereas correlations in warmth and moisture were captured using interaction terms. We did not explicitly model increased atmospheric carbon dioxide (CO_2_) impact as CO_2_ is (1) highly correlated with time [11] and further (2) several lines of investigations [37] show that the science of CO_2_ effect on crop yields is not settled. Impacts from changes in other variables were beyond the scope of this study and should be considered in future studies.

**Fig 2:**
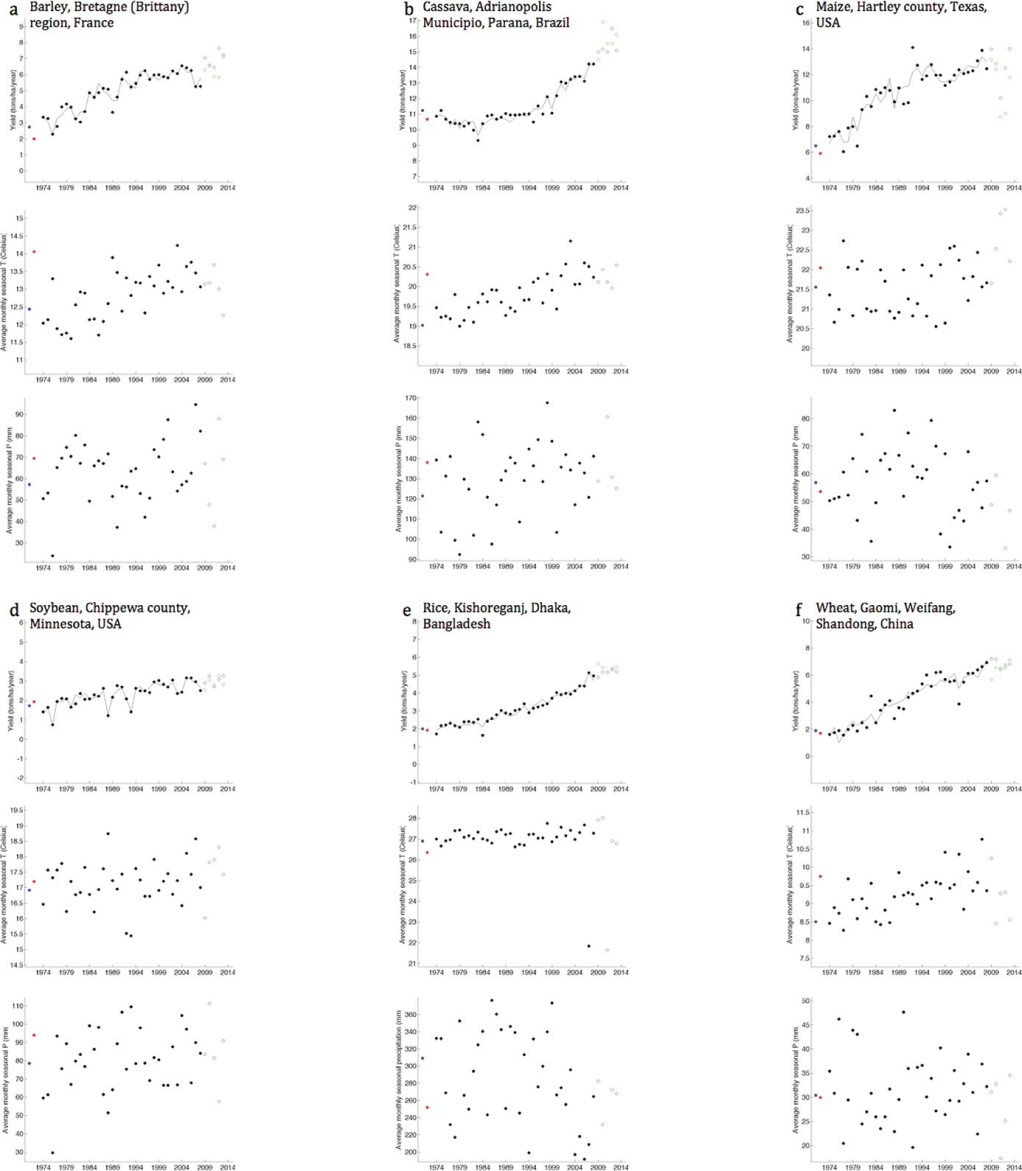
Six examples showing construction of the regression model relating observed yield (top panels) to the independent variables (middle and bottom panels–only the seasonal average observation is plotted) for example crops and political units (filled black circles) over 35 years 1974 to 2008. The models were then used for predicting yields for historical (filled blue circles) and current (filled red circles) climate conditions with time terms switched off as we are only interested in the difference to yield from difference in climate. Out-of-sample predictions do not occur as the historical and current conditions are bounded within the training weather conditions (middle and bottom panels for seasonal conditions). Yield predictions for individual years 2009 to 2013 (out of sample) are shown in open green circles and observed yield in open black circles with 5 year average error reported as follows for the specific political unit (noted in the figure) in a country: (a) barley (France) -9.3% error, (b) cassava (Brazil) 2.4% error, (c) maize (USA) -13.3% error, (d) soybean (USA) -5.3% error, (e) rice (Bangladesh) 18.4% error, and (f) wheat (China) -6.2% error.

However, high model R^2^ values, and the Gaussian nature and white noise of model residuals (indicating that residual errors were normally distributed and not auto-correlated), and cross validation against the yields of 2009 to 2013 showing low errors show that models were robust enough to answer the questions on mean precipitation and temperature change on crop yields (Fig 2; S5–S8 and S11 Figs and S2, S3 and S6 Tables).

The global summary of the mean climate changes impact on crops contained in the AR5 [2] report uses country-, regional-, and farm-level studies. These studies varied across methods, scales, and time periods and were for the top four global crops–maize, rice, wheat and soybean. Consequently a range of impacts was found; the rice and soybean 10^th^ to 90^th^ percentiles estimates covered both positive (benefit) and negative (reduction) yield impacts from climate trends. Similar multi-method analyses provide conflicting results across regions and crops [38] though recent analysis in wheat that compared point and grid based crop model simulations, with statistical regression approach, were consistent [11].

We tracked ~20,000 political units globally for 10 crops, providing more detail on the spatial resolution and a larger number of crops than previously studied [3, 11, 14, 19]. Further, this is the first observational global study reporting the impact of current climate change on the yields of the top ten global crops (Fig 1, Table 1), and six of them, barley, cassava, oil palm, rapeseed, sorghum and sugarcane are the next important global crops after maize, rice, wheat, and soybean for daily dietary calories. Some such as cassava and sorghum are a major source of calories in food insecure regions (~3.5% and ~3.4% of total food calories provided respectively). Sugarcane, oil palm and rapeseed are important commodity crops with the latter two having greatly expanded in recent decades. Another implicit advancement in our report is from computing the harvested area weighted weather statistics from reported harvesting information each year at the unit of the study ~20,000 political units.

We find a range of impacts of mean climate change on crop yields (Fig 1, Table 1 and S4 Table) and production in different regions. We found that crop yields across Europe, Sub-Saharan Africa and Australia had in general decreased because of climate change, though exceptions are present. Similar variations are seen in other crops and regions all over the world. They are indicative of the underlying variations of agronomic growing conditions that range from the agro-meteorological to crop management. Precipitation variations for example are much heterogeneous in their trends (S3 and S4 Figs) and thus captured well using time series analysis per political unit compared to the more homogeneous patterns of temperature trends (S1 and S2 Figs).

Our analysis focuses on historical precipitation and temperature change impacts on crop production and food security. Future studies should explore impacts from extreme temperature changes (for example determining thresholds [39, 40] and exposure to killing degree days [41]), extreme precipitation impacts [42] both historically, as well as for the expected future warming [43] and intensification of the hydrological cycle for larger number of crops as well as for livestock. Various agronomic changes and advancements can also mask climate change adaptation measures: irrigation expansion can occur for stabilizing crop production, expansion into new areas, or for double cropping in the dry season; but they can also serve as a measure to counter the effects of extreme heat [44]. Crop planting dates can advance in colder regions of the world as agronomic techniques advance but also because climate is getting warmer on average [45]. These nuances should be explicitly modeled in future studies to tease out such individual contributions. Other related variables that could be considered in future global studies are radiation use efficiency changes [46], weed, pest and pathogen infestation changes [47], soil moisture variability on crop productivity [48–49] and other important biophysical changes to determine their relative contributions to crop yields at the local to the global scale. Changes in both current and future variability in climate [43] and changes in future extreme events [39–42] need further analysis as well as the changing strategy of farmers especially in globally traded commodity crops. Lastly, crop yields and production are not only impacted from climate change, but also drive climate change [33].

Our findings here illustrate that climate change has potentially already affected global production of the ten largest crops and the production of consumable food calories in specific countries and globally. The approach used here complements the long-range projections, avoids many of the challenges faced by process-based models, and analyzes a broader set of crops. Although recent climate change has likely reduced overall consumable food calories in these ten crops by ~1% (or ~0.5% across all food calories), there is much variability among crops and regions. These findings can be used to target interventions for adaptation to climate change through better management, crop breeding, and switching crops as climate continues to evolve.

## Methods

### Data

We studied the top ten global crops from around the world where they are commonly harvested. Not all crops are harvested everywhere and each year.

To conduct the study we used two datasets 1) climate and weather, and 2) crop yields and harvested areas. Climate and weather variables were all derived from the Climate Research Unit (CRU) TS4.01 [18] dataset and its previous data versions have been commonly used in previous studies [13, 16, 20]. The CRU TS4.01 data is a major upgrade and supersedes all previous versions of the CRU data. We mapped the half-degree CRU derived climate and weather information to the crop harvested areas of each political unit (S1 Text section 1.1).

We carried out two major revisions to our crop data that were accessed from public sources [16] and links to the sources are provided in S1 Text section 1. We increased the spatial resolution tracked to ~20,000 political units, and from four major crops being tracked–maize, rice, wheat, and soybean–to tracking the top ten global crops–barley, cassava, maize, oil palm, rapeseed, rice, sorghum, soybean, sugarcane, and wheat. Further details of the crop data and the countries now being tracked at the sub-national levels (one or two levels below the national scale) are provided in S1 Text section 1. The new political units were mapped using information from the public source GADM (https://gadm.org/).

Inclusion of sub-national information enables capturing geographical differences, which is missed when studies are restricted to country level political units, especially applicable in cases dealing with geographically large countries. We could not entirely remove this challenge i.e. countries such as Kazakhstan were studied as a single political unit, but in 86 large countries (S1 Text) we conducted the study at one to two administrative divisions down from the country level.

To show the effects of studying large countries as a single unit versus sub-national analysis consider the impact of climate change on rice in Cambodia and Malaysia. When analyzed at the country level, rice yield change of -0.03, and +0.15 tons/ha/year was found for Cambodia and Malaysia respectively. Analysis conducted at the subnational scale when summarized to the country level however shows -0.12, and -0.06 tons/ha/year rice yield change for Cambodia and Malaysia respectively. The difference is because when studied at subnational level we are able to isolate the impacts among regions. In the case of Cambodia in the rice bowl central plains region (like Kampong Thom district) and surrounding districts large rice yield losses occur. In the southern districts along the Gulf of Thailand (districts like Koh Kong) climate change benefitted rice yields, but the gains were not enough to overcome the losses elsewhere, leading to overall national level rice yield losses. Similar effect of geography is seen in Malaysia between the eastern (gains in rice yield) and western parts (losses in rice yields) of peninsular Malaysia on the two parts of the natural divide of the Titiwangsa Range; this subnational signal can only be captured through sub-national high-resolution analysis. Thus where possible higher resolution analysis will lead to more precise results.

### Analysis

Our statistical model is a 15-parameter equation, relating crop yields to weather variables at each political unit and of the form:

$$crop\;yield=\alpha_{1}t+\alpha_{2}t^{2}+\alpha_{3}sP+\alpha_{4}sT+\alpha_{5}sP^{2}+\alpha_{6}sT^{2}+\alpha_{7}sP*sT+\alpha_{8}sP^{2}*sT^{2}+\alpha_{9}aP+\alpha_{10}aT+\alpha_{11}aP^{2}+\alpha_{12}aT^{2}+\alpha_{13}aP*aT+\alpha_{14}aP^{2}*aT^{2}+k+\varepsilon$$ (1)

where, t = time (year). The time term is included to account for technological/management changes. We have linear (t) and squared time (t^2^) terms to account for slow and rapid changes respectively as similarly used in previous studies [16, 19]. α_1_ and α_2_ are the coefficients associated with the linear and squared time terms respectively. Time terms account for omitted variable bias. sP and sT are terms to account for the contribution of gradual/linear fluctuations in the main crop growing season average precipitation (temperature) conditions on crop yield; α_3_ and α_4_ are respectively the coefficients associated with the seasonal precipitation and temperature. P and T respectively represent precipitation and temperature. sP^2^ and sT^2^ are terms that account for the contribution of extreme/quadratic seasonal precipitation and temperature conditions respectively to crop yields. α_5_ and α_6_ are the coefficients associated with these two terms. The contribution of the interaction between linear and extreme seasonal precipitation and temperature to crop yield at the political unit is from the sP*sT and sP^2^*sT^2^ terms respectively; α_7_ and α_8_ are the coefficients associated with these two terms. aP and aT terms are similar to the seasonal sP and sT terms respectively, but for capturing the contribution of annual (one year prior to harvest) weather conditions to yields; α_9_ and α_10_ are the coefficients associated with these two terms. Similarly we have terms aP^2^ and aT^2^ to account for contribution of annual extreme weather to yields, and annual interaction terms aP*aT and aP^2^*aT^2^. Coefficients associated with them are α_11_, α_12_, α_13_ and α_14_. Annual terms account for antecedent weather conditions, secondary, and third season crops, and staggered crop production, and is similar to previous modeling setup [16]. The constant of the regression is k and ε is the error term. Each political unit had this form of the model.

Setting up our regression as time series analysis allows us to more accurately capture the effect of precipitation variations. This is because precipitation variations are highly heterogeneous and previous attempts that used panel regressions are less sensitive to precipitation variations [50]. The setting up of this form of the model is uniquely to answer only questions on climate trends/mean climate change impact on crop yields for historical and current conditions. To answer other questions such as identifying temperature thresholds beyond which crops have severe yield losses other forms of model specifications are required, such as an eighth-order polynomial function of temperature [39].

A linear fit using observed weather variables (Fig 2) with observed yield (Eq 1 above) was thus constructed for each crop and political unit (the fitting method is QR decomposition). The model was next tested for significance at the p < 0.05 levels (ANOVA F-statistics; NULL model is the average yield). If the linearly fitted model was significant it was then used to conduct predictions of yield for historical and current climate condition (which are in-sample predictions, as historical and current climate condition are within the range of observed weather–Fig 2). The difference in the predicted yield is the likely observed impact on crops yield due to mean climate change/climate trends at the level of the political unit (see more elaboration in S1 Text sections 2). Residuals were found to be normally distributed and not autocorrelated (S7 Fig), with overall R^2^ of the models generally greater than 0.8 (S3 Table, S8 Fig), indicating that the models could be used to answer questions on climate change. The lowest (minimum) R^2^ value in the linear regressions retained for this analysis from anywhere globally was in wheat (0.42 for the political unit of Ryazan oblast in the Central Federal region of the Russian Federation; the root-mean-squared error here was 0.4 tons/ha whereas the observed average yields are ~2.7 tons/ha/year). In all these cases, the goodness of fit test rejects the hypothesis of a lack of fit to the data (at the p = 0.05 level). Cross validation of model yield predictions against observations (2009 to 2013) showed low prediction errors (S2 Table).

We do not report the Akaike or Bayesian Information criteria (AIC/BIC) here since these merely reinforce the goodness-of-fit results. Coefficient confidence intervals may be obtained by routine methods, and are not reported here for brevity, but are available. Low model R^2^ that exists in some regions for some crops such as in Ryazan oblast for wheat should be borne in mind when interpreting results for those areas. Consistent with this approach, we restrict use of our models to climate conditions within the bounds of the observed weather conditions under which the models were constructed i.e. we conducted within-sample predictions and avoided out-of-sample predictions (see S1 Text section 2 and S5 and S6 Figs). Predictions being from statistical analysis, these are not deterministic, and the globally harvested area averaged model Mean Squared Errors (MSE) are provided in S6 Table and mapped in S11 Fig for each crop. Sensitivity to individual model coefficients (sensitivity = coefficient/change in variable) connected to seasonal temperature, squared seasonal temperature, precipitation and squared seasonal precipitation are provided in S12 Fig for maize, rice and wheat that shows how differently each term behaves; the temperature only and precipitation only change impact is provided in S9 and S10 Figs and the full impact in Fig 1. The model formulation per crop and political unit that we determined are provided in S7 Table and the corresponding maize, rice, soybean and wheat yield data for the study period is provided in S8 Table.

We have used a relatively simple statistical framework to tease out the main effects of changed climatic conditions on crop yields. A potential limitation of the analysis presented is that regression models were separately estimated within each geographical unit, thus we ignore spatial auto-correlations. Given our spatial scales–even though finer scaled compared to the country scale–each political unit nevertheless are hundreds to thousands of squared kilometers spatially–diffusion of agronomic knowledge and other socio-economic variables i.e. spatial autocorrelation effects would be relatively limited compared to analyses at much more finer spatial scales such as at village levels. Future studies should explore spatial and temporal multi-scale effects [50]; we do not consider potential physical interaction between the explanatory variables at different scales in different geographical units. We would also like to caveat against interpreting our regression-based analysis as causality. Needless to say, detailed studies using technically more complex statistical models, including causal models, and more extensive model diagnostics, is needed. Ignoring spatial autocorrelation does not necessarily bias the final results, however inclusion of such effects may improve the precision of the estimates. On the other hand, spatio-temporal statistical models can be computationally and mathematically very challenging. Future publications should try to address spatio-temporal dependency, diagnostics associated with statistical modeling and other issues mentioned above in global-scale studies.

For computing production change we multiplied the climate change driven yield change with the 5-year average harvested areas (2004–2008); for computing percentage yield change with respect to recent yields we similarly computed a 5-year average yield (2004–2008) at each political unit. Country- and global-scale numbers are similarly all harvested area weighted. Finally we determined the change in food calories in a country as the sum of all of individual crop impacts out of the maximum of ten crops studied; most countries did not harvest all ten crops. Details are provided in S1 Text.

### Mapping

After determining the yield change at the political unit due to climate change we mapped the result back at the political unit level. Each political unit contains grid cells that harvested a crop and we assign all these grid cells the same yield change impact. The results are thus valid only at the scale of the political unit for which data were available. In cases where sub-national data were not available there is a marked difference at the state or country borders. For example, Kazakhstan was studied at the country level only and therefore results are valid only at that scale and the contrast with for example the Russian Federation boundary are from mapping. In regions of the world where higher resolution information was available boundary effects are not present e.g. US-Mexico boundary for maize, India-Nepal-Bangladesh boundary for wheat or rice.

## Supporting information

S1 Text Additional data and statistical analysis information. (PDF)

S1 Fig Seasonal monthly climatological temperature change map for the ten crops by political units (PU). (PDF)

S2 Fig Annual monthly climatological temperature change map for the ten crops by PU. (PDF)

S3 Fig Seasonal monthly climatological precipitation change map for the ten crops by PU. (PDF)

S4 Fig Annual monthly climatological precipitation change map for the ten crops by PU. (PDF)

S5 Fig Three examples (a), (b), & (c) when model cannot be used (plotted in the maps of Fig 1 in the main text as white colored areas).
(PDF)

S6 Fig Weather information used for constructing the regression model for example crop and country cases (solid black filled circles). The models were then used for historical (open blue circles) and current (open red circles) climate conditions. Out-of-sample predictions do not occur as the historical and current conditions are bounded within the training weather conditions. (a) Maize (USA), (b) Rice (China), (c) Wheat (India), (d) Soybean (USA) (e) Maize (Mexico) and (f) Maize (China). Training weather data size is (N) X (35) and shown figures are for seasonal monthly conditions. Annual monthly conditions showed similar results (not shown).
(PDF)

S7 Fig Areas with statistically significant white noise error (at p = 0.01 level, red colored regions, 0% to 8% of all studied regions depending on the crop) as determined from Ljung-Box Q-Tests for autocorrelation in the residuals per crop and political unit. (PDF)

S8 Fig Coefficient of determination of the model for the ten crops mapped by PU. (PDF)

S9 Fig Change in crop yield due to only temperature climatological change (holding the precipitation variables at historical levels). (PDF)

S10 Fig Change in crop yield due to only precipitation climatological change (holding the temperature variables at historical levels). (PDF)

S11 Fig Maps of model Mean Squared Errors (MSE) (tons/ha/year). (Note the variable legends which goes from zero to about half of global averaged yields–S4 Table).
(PDF)

S12 Fig Sensitivity maps to seasonal P, T, P^2^ and T^2^ for maize, rice and wheat where sensitivity = coefficient / change. (PDF)

S1 Table Climatological change in temperature and precipitation (values are averaged over all harvested croplands including those with insignificant climate change impact or unstudied due to data limitations). Change is the difference between monthly average current and historical value.
(PDF)

S2 Table Harvested area weighted average model yield prediction errors for 2009 to 2013 globally (percentage off from observations). (PDF)

S3 Table Production weighted coefficient of determination averaged globally per crop. (PDF)

S4 Table Change in crop yields and kilocalorie change summarized at the country level. (XLSX)

S5 Table Percentage yield changed w.r.t historical global scale yield. (Yields under current T or P change are only over areas where change was detected and historical yields are over all cropped areas. Computed changes are not additive).
(PDF)

S6 Table Globally averaged Mean Square Error (MSE) (tons/ha) averaged over the harvested areas studied. Current yield refers to averaged yields over areas reported over years 2004–2008. (PDF)

S7 Table Model determined per crop and political unit. (XLSX)

S8 Table Yield data for the political units studied (maize, rice, soybean, and wheat). (XLSX)
