# Supplementary material for: Climate change has likely already affected global food production
Source: PLoS One. 2019 May 31;14(5):e0217148. doi: 10.1371/journal.pone.0217148 (PMC6544233; doi:10.1371/journal.pone.0217148)
Supplement: S1 Text — (PDF) [file pone.0217148.s001.pdf]

S1 Text Additional data and statistical analysis information

Climate change already affects global food production

Deepak K. Ray[*,1], Paul C. West[1], Michael Clark[2,3], James S. Gerber[1], Alexander V. Prishchepov[4], Snigdhansu Chatterjee[5]

Affiliations:
[1]Institute on the Environment (IonE), University of Minnesota, Saint Paul, Minnesota, USA
[2]Department of Natural Resources Science and Management, University of Minnesota, Saint Paul, Minnesota, USA
[3]now at the Department of Population Health, University of Oxford, Oxford OX3 7LF, UK
[4]Department of Geosciences and Natural Resource Management, University of Copenhagen, Denmark
[5]School of Statistics, University of Minnesota, Minneapolis, Minnesota, USA

([*]Email: dray@umn.edu)
Telephone: 612-6264029

1. Data

1.1 Climate and weather data

We used the temperature and precipitation information from the Climate Research Unit (CRU) TS4.01 (*1-4*) dataset for developing the weather and climate metrics (https://crudata.uea.ac.uk/cru/data/hrg/cru_ts_4.01/). The CRU data is built using station reported observations with additional analyses for gridding the observations to a half-degree spatial resolution. Global weather datasets of direct observations spanning a long time scale are few, and when the CRU data was cross-compared in previous studies (*5*) with other global weather observational datasets (i.e. University of Delaware Terrestrial Air Temperature and Precipitation dataset in [*5*]) it was found that the results were similar. Furthermore CRU data has been used commonly in previous investigations (*6-9*).

The grid cells within each political unit (the final unit of analysis is the political unit) were at 5 min spatial resolution at which crop harvest extent is reported; the CRU TS4.01 data was at a coarser half a degree spatial resolution. We assigned each of the 5-minute (~10 km X 10 km) crop grid cells that were contained within a half-degree CRU grid cell (~110 km X 110 km), and reporting crop harvests, the CRU reported weather value similar to previous methods (*6*). In other words we re-gridded the CRU data from half degree to five minutes over all crop-harvested areas without any additional statistical or dynamical downscaling, following a nearest-neighbor approach. We did this to compute the harvested area weighted temperature and precipitation information for the entire political unit each year using the information on annual harvested areas at the 5 min spatial resolution; this was the computed political unit's harvested area weighted monthly average weather in a given year. Only those coastal 5 min grid cells with nearest half degree CRU grid cell over water bodies (and hence with fill values) were not included in computing the political unit's average weather; all other grid cells with harvested area information and non-filled weather information with center within the boundaries of the political unit was used. The process was repeated for each month and year and if for any year the political unit did not report the harvest of a crop (i.e. yield and harvested area information was missing), analysis was stopped for the political unit.

Alternate sources of long-term data such as using station observations, and reanalysis datasets such as NCEP have their own shortcomings (spatial resolution, time frame) and errors (site changes, instrument failure, calibration issues, incorrect reporting). An analysis of simulated crop yield potential at four selected sites each in the United States (for maize), Germany (for wheat) and China (for rice) however found wide variations in the simulated yield potential (not the focus of this study) on account of input weather differences (*10*) (though the gridded data was simplistically downscaled in the study). The CRU dataset grids station data after conducting data accuracy checks and utilizing known statistical methods for estimating information in data sparse regions (*1-4*). Using station data directly in

this study would require building a new gridded dataset or statistically up-scaling the station data globally, essentially reworking the steps of CRU data. The NCEP data (Reanalysis-2) is not long enough for our study (available from 1979) and if used would require downscaling the data (spatial resolution is 2.5° X 2.5°). It was thus best to directly use observations as closely as possible, i.e. use the CRU dataset.

We build the relationship between harvested crop yields and weather using both average seasonal and average annual weather conditions. Seasonal average temperature and precipitation was computed from the political unit's monthly observed weather. For determining the season average precipitation and temperature one needs to know the average crop planting and harvest date. We accessed a gridded crop planting and harvested dataset (*11*) and its major revision (*6*). Note that the CRU data reports monthly numbers. So even though the average crop planting and harvest exact date information was available for the main crop season we used the month of the average start of planting and the month of average end of harvesting as the starting and ending months respectively from the weather data and the average of all these months (both months included) to compute the average harvested area weighted temperature and precipitation for the entire season.

Additionally annual average precipitation and temperature weather was computed to account for (a) antecedent conditions and (b) to introduce the effect of weather on the yields of second and third season crop growing conditions, or staggered crop production systems that is common in the warmer parts of the world but was undifferentiated in our crop and crop calendar datasets. Following [*6*] annual refers to one year prior to the main crop harvest month (inclusive). Further studies should look at the impacts from mean climate change at different stages of crop growth (*12-14*), which however would require more precise and time varying (year-to-year variation) crop calendar information globally, which is not a trivial task (*15*).

In order to compute the impact of temperature and precipitation climatic change on crop yields we also needed to build climatological seasonal and annual, temperature and precipitation information. For this we used the same CRU TS4.01 data (that spans the period 1901 to 2010) and developed first a historical 30-year average temperature and precipitation ending in the year 1974 (1945 to 1974) for seasonal and annual historical climate per political unit. Once the annual and seasonal weather information was built per year we linearly regressed the 1974 to 2008 seasonal and annual temperature and precipitation conditions to determine the trend in weather. We added the product of the trend and 35 years, to the historical climate to build a synthetic current climatology of temperature and precipitation variables (see variables in section 2).

The difference of current and historical climate shows that averaged globally over the crop harvested areas, in all the ten crops, both the seasonal and annual temperatures increased (S1 Table) though with significant variations among regions (S1 and S2 Figs). Precipitation changes over all reported crop harvest areas with

weather information however showed both increases and decreases depending on the crop and region (S1 Table, S3 and S4 Figs). Note that even though temperature and precipitation may have changed in a political unit, it may not necessarily have led to statistically significant changes in crop yields (Fig 1 – white colored areas).

Utilization of fine resolution annual crop harvest information, in constructing annual harvested area weighted weather information, reduces the chance of introducing weather data related errors in building the regression models compared to previous studies that used static crop harvest information of circa 2000 (*5, 16*). Another source of error in previous reports was from including incorrect grid cells in the study on account of inaccuracies / incomplete knowledge of where crops were harvested (*16*), missing out on where they were actually harvested in some regions and reporting them as harvested in other regions whilst they actually were not. The third source of error that propagated in previous reports was from errors in the crop calendar (*10*) used, with incorrect planting and harvest dates in certain crops and regions, but in this report we used a more accurate though static crop calendar (*6*). How these sources of input data inaccuracies and methodology (see S1 Text section 2) propagated in previous reports and impacted the final outcome is unknown but could explain why there were differences with the current study in maize, rice, wheat, and soybean in certain countries / regions (e.g. maize in southern Africa). Further previous studies (*5*) did not provide any information on crop impacts in specific countries e.g. maize in Mexico, rice in Myanmar, wheat in Iran, and soybean in India that we now provide. As data development is a slow painstaking process future studies should strive to quantify the impact of climate trends / mean climate change more accurately from using even better quality data.

1.2 Crop data

The crop yield and area harvested information came from a major revision of the dataset noted in our previous reports using similar methods (*6, 17*). Two major improvements were done to the data and used for this analyses: (1) we increased the number of crops in our dataset from the top four global crops: maize, rice, wheat and soybean to include the six next important global crops (in terms of global calories harvested circa 2005 (*18*)): barley, cassava, oil palm, rapeseed, sorghum and sugarcane. These top ten crops together contributed to ~82.5% of total calories harvested (valid at circa 2005 (*18*)). (2) We also increased the spatial resolution tracked in this study, to ~20,000 political units tracked for all ten crops from ~13,500 political units tracked (*6, 17*). This represents a 1.5X increase in spatial resolution and a 2.5X increase in crop information which itself (*6, 17*) was a ~50X increase in spatial information from previous national scale studies. The additional ~6400 political units tracked were in the following countries (within brackets are given the number of political units (PU) tracked and data source):

1. Afghanistan (32 provinces – Afghanistan Statistical Yearbooks http://cso.gov.af)
2. Angola (18 provinces – National CountrySTAT http://www.fao.org/in-action/countrystat/national-countrystat-sites/en/)
3. Bangladesh (64 districts / zilas – Yearbook of Agricultural Statistics of Bangladesh www.bbs.gov.bd)
4. Benin (76 communes – National CountrySTAT http://www.fao.org/in-action/countrystat/national-countrystat-sites/en/)
5. Bhutan (20 districts / dzongkhags – Ministry of Agriculture and Forests http://www.moaf.gov.bt/)
6. Botswana (30 sub-districts – National CountrySTAT http://www.fao.org/in-action/countrystat/national-countrystat-sites/en/)
7. Brunei* (4 districts / daerah-daerah – International Rice Research Institute http://ricestat.irri.org:8080/wrs)
8. Burkina Faso (13 regions – National CountrySTAT http://www.fao.org/in-action/countrystat/national-countrystat-sites/en/)
9. Burundi (17 provinces – National CountrySTAT http://www.fao.org/in-action/countrystat/national-countrystat-sites/en/)
10. Cambodia* (25 provinces / khaets – International Rice Research Institute http://ricestat.irri.org:8080/wrs)
11. Cameroon (10 provinces – National CountrySTAT http://www.fao.org/in-action/countrystat/national-countrystat-sites/en/)
12. China (2408 counties / district, autonomous county, county city http://data.stats.gov.cn/easyquery.htm?cn=C01
    http://www.data.ac.cn/zrzy/DH36.asp?name=%CE%DE&pass=%CE%DE&danwei=%CE%DE
    Li et al. (2016) Patterns of Cereal Yield Growth across China from 1980 to 2010 and Their Implications for Food Production and Food Security, PLOS ONE, https://doi.org/10.1371/journal.pone.0159061)
13. Democratic Republic of Congo (38 sub-regions / sous-regions – National CountrySTAT http://www.fao.org/in-action/countrystat/national-countrystat-sites/en/)
14. Ethiopia (11 regional states and chartered cities / kilils – National CountrySTAT http://www.fao.org/in-action/countrystat/national-countrystat-sites/en/)
15. Gambia (6 divisions and independent city – National CountrySTAT http://www.fao.org/in-action/countrystat/national-countrystat-sites/en/)
16. Ghana (10 regions – National CountrySTAT http://www.fao.org/in-action/countrystat/national-countrystat-sites/en/)
17. Guinea Bissau (9 regions and autonomous sector – National CountrySTAT http://www.fao.org/in-action/countrystat/national-countrystat-sites/en/)
18. India (2340 tehsils / taluks – Directorate of Economics and Statistics https://eands.dacnet.nic.in/ and http://vdsa.icrisat.ac.in/vdsa-database.aspx)
19. Indonesia (444 regency and municipality / kabupaten and kotamadya – International Rice Research Institute http://ricestat.irri.org:8080/wrs;

World Bank https://databank.banquemondiale.org/data/source/indonesia-database-for-policy-and-economic-research/preview/on)

20. Ivory Coast (19 regions – National CountrySTAT http://www.fao.org/in-action/countrystat/national-countrystat-sites/en/)
21. Japan (47 prefectures, circuits, metropolis / ken, do, to – Statistics Department, Minister's Secretariat, Ministry of Agriculture, Forestry and Fisheries)
22. Kenya (8 provinces / mkoa – National CountrySTAT http://www.fao.org/in-action/countrystat/national-countrystat-sites/en/)
23. Laos (18 provinces / khoueng – International Rice Research Institute http://ricestat.irri.org:8080/wrs)
24. Lesotho (10 districts – National CountrySTAT http://www.fao.org/in-action/countrystat/national-countrystat-sites/en/)
25. Liberia (15 states – National CountrySTAT http://www.fao.org/in-action/countrystat/national-countrystat-sites/en/)
26. Madagascar (110 districts / fivondronana – National CountrySTAT http://www.fao.org/in-action/countrystat/national-countrystat-sites/en/)
27. Malawi (28 states – National CountrySTAT http://www.fao.org/in-action/countrystat/national-countrystat-sites/en/)
28. Malaysia* (13 states – International Rice Research Institute http://ricestat.irri.org:8080/wrs)
29. Mali (9 regions – National CountrySTAT http://www.fao.org/in-action/countrystat/national-countrystat-sites/en/)
30. Mozambique (11 provinces / provincia– National CountrySTAT http://www.fao.org/in-action/countrystat/national-countrystat-sites/en/)
31. Myanmar (15 states, divisions, union territory / pyine, yin, union territory - Central Statistical Organization Nay Pyi Taw, Myanmar)
32. Namibia (13 states – National CountrySTAT http://www.fao.org/in-action/countrystat/national-countrystat-sites/en/)
33. Nepal (75 administrative zones / anchal – Ministry of Agricultural Development, statistics division, Kathmandu Nepal)
34. Niger (8 departments – National CountrySTAT http://www.fao.org/in-action/countrystat/national-countrystat-sites/en/)
35. Pakistan (141 districts – Ministry of Economic affairs and Statistics http://www.pbs.gov.pk/content/agriculture-statistics)
36. Philippines (81 provinces / lalawigan / probinsya – International Rice Research Institute http://ricestat.irri.org:8080/wrs)
37. Russian Federation (75 oblasts - Rosstat Statistical Yearbooks "Agriculture in Russia" & USSR CSD National Economy of RSFSR. Statistical yearbooks. Moscow: Statistika, Central Statistical Department)
38. Swaziland (4 districts– National CountrySTAT http://www.fao.org/in-action/countrystat/national-countrystat-sites/en/)
39. Taiwan (national – FAOSTAT http://www.fao.org/faostat/en/#data/QC)
40. Tanzania (183 districts– National CountrySTAT http://www.fao.org/in-action/countrystat/national-countrystat-sites/en/)

41. Togo (5 regions – National CountrySTAT http://www.fao.org/in-action/countrystat/national-countrystat-sites/en/)
42. Zambia (10 provinces – National CountrySTAT http://www.fao.org/in-action/countrystat/national-countrystat-sites/en/)

(* denotes countries where only rice was studied at the subnational level; other crops were studied at the national level due to data limitations. Country level data was from FAOSTAT http://www.fao.org/faostat/en/#data/QC. All data accessed variously between the years 2015 and 2017.)

Most of the above mentioned 42 countries were tracked in our previous reports (*6, 17*) only at the country level such as in Burkina Faso, or not tracked as in Taiwan. Where we tracked at the sub-national (first level such as 14 zones in Nepal) we now track at the next level (below the state / provincial level) as at 75 anchals in Nepal, or the 47 prefectures in Japan.

We continued to track the 44 other countries at the first- and second- political units level below the national level as reported in our previous reports (*6, 17*) such as in the 5504 municípios of Brazil, and in the 3148 counties of United States. Thus altogether 86 countries were tracked at one or two levels below the country level (the FAO reported level) globally in this study. For the remainder of the countries such as Egypt or Nicaragua we continue to track only at the national level (i.e. we use the FAO numbers only) for the yield information due to data limitations, and we do not have any additional subnational skill, except for the location of croplands; we assign uniform crop yields in all the cropland grid cells (*19*) in these countries though harvested amounts varied – e.g. zero where cropland was zero. For the former Soviet states such as Russian Federation we are now able to track reported yield information prior to the breakup (pre-1991) of the Soviet Union annually.

The sum of country reported sub-national harvested areas were scaled to match the FAO reported country numbers each year. Total production was similarly matched to FAO reported numbers in each crop and year. Yield is the derived quantity after any correction required for harvested areas and production quantities. Scaling was done (as previously e.g. [*17*]) in two steps if we tracked a country at the county / district level (sub sub-national level). First we scaled up or down county reported numbers such that the sum of the county / district level data matched the state / provincial reported totals and then the total of the states / provinces were scaled up or down to match the FAO reported national values. If the states were scaled, then the scaling factors were applied down to the county / district levels so that if one summed up the county / district values directly across the entire country the result would match the FAO reported number. If we tracked only at the states / provinces level then the scaling of state reported numbers was done so as to match the state totals to the FAO reported values. Following [*17*] when sub-national data became unavailable before or after a specific year, we used the nearest 5 years average data to determine proportions, and apportion the information available at the next coarser spatial unit level to this finer scale political unit.

The crop statistics database provides information on harvested areas for each of the ten crops: barley, cassava, maize, oil palm, rapeseed, rice, sorghum, soybean, sugarcane and wheat. Total harvested area for a political unit was distributed as a function of the fraction of cropland within each 5 min grid cell in the political unit. Within a political unit, however at each 5 min grid cell the crop yields are assumed constant over the harvested areas. The unit of analysis is not 5 min but the political unit. The yield and harvested information does not distinguish between the primary, second and third crop seasons such as rice grown in southern China, southern India, or in Bangladesh, in double and triple cropping systems. Crops such as wheat that are grown as winter or spring wheat as in the United States or Russian Federation are not distinguished, though the primary crop season was known from the crop calendars. Sugarcane primary and *ratoon* crops are also not distinguished. Staggered planting, leading to staggered harvests, over a period of weeks to months and are also not distinguished but common in tropical regions. Other distinctions such as different cultivars, upland and lowland crops, GMO and non-GMO crops, irrigated and non-irrigated crops are not distinguished either but may have differential climate change impacts. To account for contributions to yield from out-off-main-season harvests spread over the year we had additional terms to capture annual conditions (section 2). Further research is required to account for these differences but not easily accomplished on account of lack of such additional dataset requirements globally.

A back of the envelope calculation would show that tracking ten crops for 35 years with two independent statistics (harvested areas and yield) at the ~20,000 political units would imply that 14 million data points were tracked around the world. In reality as the crops are not harvested at each political unit, and lack of long term crop data eliminated other political units, we report at fewer political units that we know continuously harvested crops. Oil palm for example is reported for ~1800 political units globally, but maize, a more widely grown crop is reported at ~12,500 political units.

Oil palm, rapeseed, soybean and sugarcane are commodity crops that have recently witnessed a surge in their harvest extent and new political units have started harvesting these crops consistently and reports available from recent years. The reporting initially often is episodic but data suppressed due to farmer privacy issues in such political units. Lack of long-term data removed such political units more commonly and in these commodity crops from our study reducing the total studied areas compared to current harvesting extent in oil palm, rapeseed, soybean, and sugarcane by ~18%, ~10%, ~18%, and ~17% respectively. In the remaining six crops the reporting was reduced by ~5%: barley (~6%), cassava (~6%), maize (~6%), rice (~4%), sorghum (~4%), wheat (~6%).

2. Statistical analysis

2.1 Avoiding out-of-sample predictions

Our modeling system avoids out-of-sample prediction as it is built using observed weather conditions in the same political unit where it is applied (S5 and S6 Figs). Consequently the historical and current climate conditions are within the range of observed weather conditions. Further, the potential for over-fitted regressions though present, application of our models being not for out-of-sample conditions (spatially or temporally) and only applied in the political unit where the model was built, we are able to capture the impact of observed mean climate change / climate trends. Over fitted models built for one set of conditions and then applied for another condition will not give correct predictions, which is never the case here.

2.2 Determining climate change impact from climate change

We used the historical climate parameters once and current climate parameters once in the modeled regression relationship and recorded the two yield responses (with time terms switched off). The difference between the two yield values is the impact of climate change on crop yield for the political unit. Note that this does not provide any sub political unit information and our results should be used as an averaged climate change impact for the entire political unit. The five year (2004-2008) harvested area weighted yield average was computed and the climate change impact reported as percentage changes to current yield (Table 1).

Yield change from holding the temperature variables at historical level (S9 Fig) and from holding the precipitation variables at historical level (S10 Fig) while allowing the other to change provides an indication of the relative importance of the temperature and precipitation changes respectively.

At each political unit we first checked whether the regression was better than a random climate model (at $p < 0.05$). Only those political units where statistically significant modeled relationships were found (54-88% of harvested areas in the ten crops globally; Table 1) we determined the impact of climate change on crop yields. On a note of caution, even though the modeled relationship is statistically significant in a political unit, the coefficient of determination (R^2) of the model could be low (S8 Fig). Generally the R^2 values were > 0.8 indicating more than 80% of the variations in yields were accounted for in the model. In regions with low R^2 readers should use our results with greater caution though averaged globally they ranged 0.76 to 0.87 depending on the crop (S3 Table).

2.3 Sensitivity to temperature and precipitation

To assess sensitivity to temperature and precipitation we used the models to determine the impact of temperature only change (S9 Fig) and precipitation only

change (S10 Fig) on crop yield. The results are not additive as there are interaction terms whose effects cannot be measured in this set up, and both precipitation and temperature impacts were not linear everywhere and impacts felt everywhere (S4 Table).

2.4 Converting change in crop production to change in consumable food calories

We also estimated the change in caloric availability from changed production on account of mean climate change. To do so, we first estimated the caloric production impact climate change had for each crop and country using crop-specific nutrient densities derived from the FAO's Food Balance Sheets (FBS) (*22*). The proportion of crop production allocated to food (direct human consumption of a crop), feed (used to produce livestock products, e.g. beef), or processing (used to produce processed goods, e.g. sugar) was then calculated for each crop and country from data available on the FBS. Net change in caloric availability of food, feed, and processing was calculated as the product of net change in caloric production and the proportion of total production allocated to food, feed, or processing, respectively. While calories allocated to food are directly available for human consumption, calories allocated to feed and processing are not. As such, we first estimated the average feed conversion ratio, or the amount of feed calories required to produce a single food calorie, for each country across all livestock products using consumption patterns reported in the FBS (*22*) and food-specific feed conversion ratios used in [*23*]. Similarly, we estimated changes in caloric availability of processed foods using crop-specific estimates of the efficiency of converting raw foods (e.g. sugar cane) into processed foods (e.g. sugar). The total change in caloric availability is thus the sum of the changes of food, animal-based food, and processed food caloric availability.

There were some data limitations in food caloric conversion from production. FAO does not provide food balance sheets for some countries such as Burundi, Democratic Republic of Congo, Bhutan, and Syria. Even though these countries have witnessed crop production changes we could not compute caloric changes. Further in a few countries while a crop was harvested, and change in crop production on account of mean climate change detected, according to FBS there was no dietary contribution, such as in barley in Australia. We thus removed these crop and country combinations when computing the impact on consumable calories from mean climate changes. Such harvested crops are likely traded internationally, which in turn varies depending on year and country, thereby distorting the changes in consumable calorie by crop and country. For example, while Australia does not consume barley calories, its export to different countries globally would distort the percentage change in consumable barley food calories by country, regions as well as globally (Table 1) though the effect will diminish as we aggregate increasingly larger spatial units.

References

1. I. Harris, P. D. Jones, T. J. Osborn, D. H. Lister, Updated high-resolution grids of monthly climatic observations – the CRU TS3.10 dataset. International Journal of Climatology **34**, 623-642 (2014)
2. M. New, M. Hulme, P. D. Jones, Representing twentieth-century space-time climate variability. Part I: development of a 1961-90 mean monthly terrestrial climatology. Journal of Climate **12** 829–856 (1999)
3. M. New, M. Hulme, P. D. Jones, Representing twentieth century space-time climate variability. II: development of 1901–1996 monthly grids of terrestrial surface climate. Journal of Climate **13** 2217–2238 (2000)
4. T. D. Mitchell, P. D. Jones, An improved method of constructing a database of monthly climate observations and associated high-resolution grids. International Journal of Climatology **25** 693–712 (2005)
5. D. Lobell, W. Schlenker, J. Costa-Roberts, Climate trends and global crop production since 1980. Science **333**, 616-620 (2011)
6. D. K. Ray, J. S. Gerber, G. K. MacDonald, P. C. West, Climate variation explains a third of global crop yield variability. Nature Communications **6**, doi:10.1038/ncomms6989 (2015)
7. B. Poulter et al., Contribution of semi-arid ecosystems to interannual variability of the global carbon cycle. Nature, **509**, 600-603 (2014)
8. R. A. Garcia et al., Multiple Dimensions of Climate Change and Their Implications for Biodiversity. Science **344**: 6183, DOI: 10.1126/science.1247579 (2014)
9. A. C. Ruane et al., Climate forcing datasets for agricultural modeling: Merged products for gap-filling and historical climate series estimation. Agricultural and Forest Meteorology, **200**, 233-248 (2015)
10. J. Van Wart, P. Grassini, K. G. Cassman, Impact of derived global weather data on simulated crop yields. Global Change Biology **19**, 3822-3834 (2013)
11. W. J. Sacks, D. Deryng, J. A. Foley, N. Ramankutty, Crop planting dates: an analysis of global patterns. Glob. Ecol. Biogeogr. **19**, 607-620 (2010)
12. E. E. Butler, P. Huybers, Adaptation of US maize to temperature variations. Nature Climate Change **3**, 68-72 (2013)
13. S. M. Gourdji, A. M. Sibley, D. B. Lobell, Global crop exposure to critical high temperatures in the reproductive period: historical trends and future projections. Environmental Research Letters **8**:2, 024041 (2013)
14. F. Tao et al., Wheat yield benefited from increases in minimum temperature in the Huang-Huai-Hai Plain of China in the past three decades. Agricultural and Forest Meteorology **239**, 1-14 (2017)
15. C. J. Kucharik, A multidecadal trend of earlier corn planting in the central USA. Agronomy Journal, **98**, 1544–1550 (2006)
16. C. Monfreda et al., Farming the planet: 2. Geographic distribution of crop areas, yields, physiological types, and net primary production in the year 2000. Global Biogeochemical Cycles **22**, GB1022, doi:10.1029/2007GB002947 (2008)
17. D. K. Ray, N. Ramankutty, N. D. Mueller, P. C. West, J. A. Foley, Recent patterns of crop yield growth and stagnation. Nature Communications **3**:1293 doi:10.1038/ncomms2296 (2012)

18. D. Tilman, C. Balzer, J. Hill, B. L. Befort, Global food demand and the sustainable intensification of agriculture. Proc Natl Acad Sci USA **108**: 20260–20264 (2011)
19. N. Ramankutty et al., Farming the planet: 1. Geographic distribution of global agricultural lands in the year 2000. Global Biogeochem. Cycles **22**, GB1003 (2008)
20. D. B. Lobell, M. B. Burke, On the use of statistical models to predict crop yield responses to climate change. Agricultural and Forest Meteorology **150**, 1443-1452 (2010)
21. W. Schlenker, M. J. Roberts, Nonlinear temperature effects indicate severe damages to U.S. crop yields under climate change. Proc Natl Acad Sci USA **106**: 15594–15598 (2009)
22. FAO 2017, Food balance sheets, http://www.fao.org/faostat/en/#data/FBS
23. D. Tilman, M. Clark, Global diets link environmental sustainability and human health. Nature **515**, 518–522 (2014)
