# Supplementary material for: Climate change has likely already affected global food production
Source: PLoS One. 2019 May 31;14(5):e0217148. doi: 10.1371/journal.pone.0217148 (PMC6544233; doi:10.1371/journal.pone.0217148)

S1 Fig Seasonal monthly climatological temperature change map for the ten crops by political units (PU).

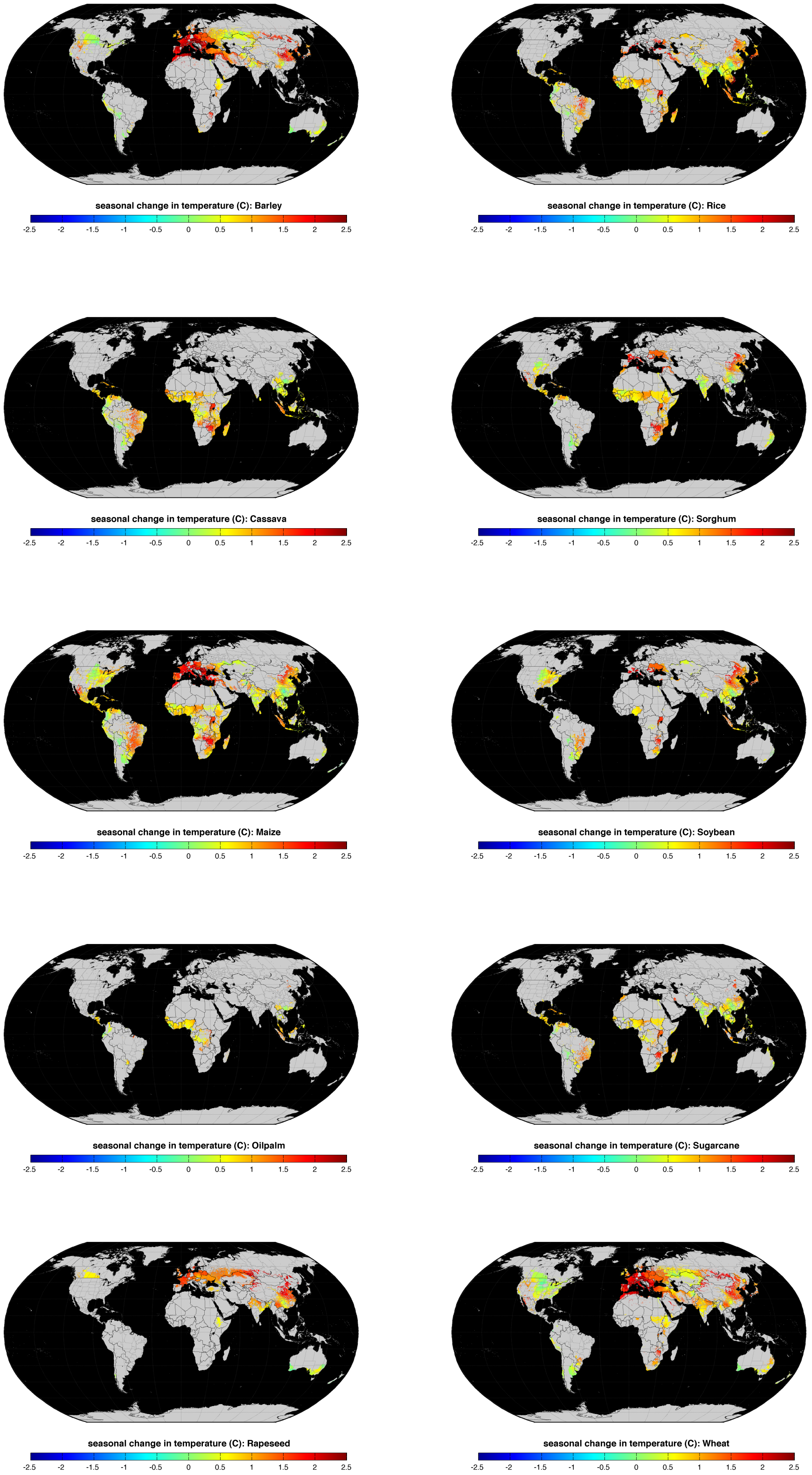

Supplement: S1 Fig — (PDF) [file pone.0217148.s002.pdf]
