# Supplementary figures and images for: Climate change has likely already affected global food production
Source: PLoS One. 2019 May 31;14(5):e0217148. doi: 10.1371/journal.pone.0217148 (PMC6544233; doi:10.1371/journal.pone.0217148)

S2 Fig Annual monthly climatological temperature change map for the ten crops by PU.

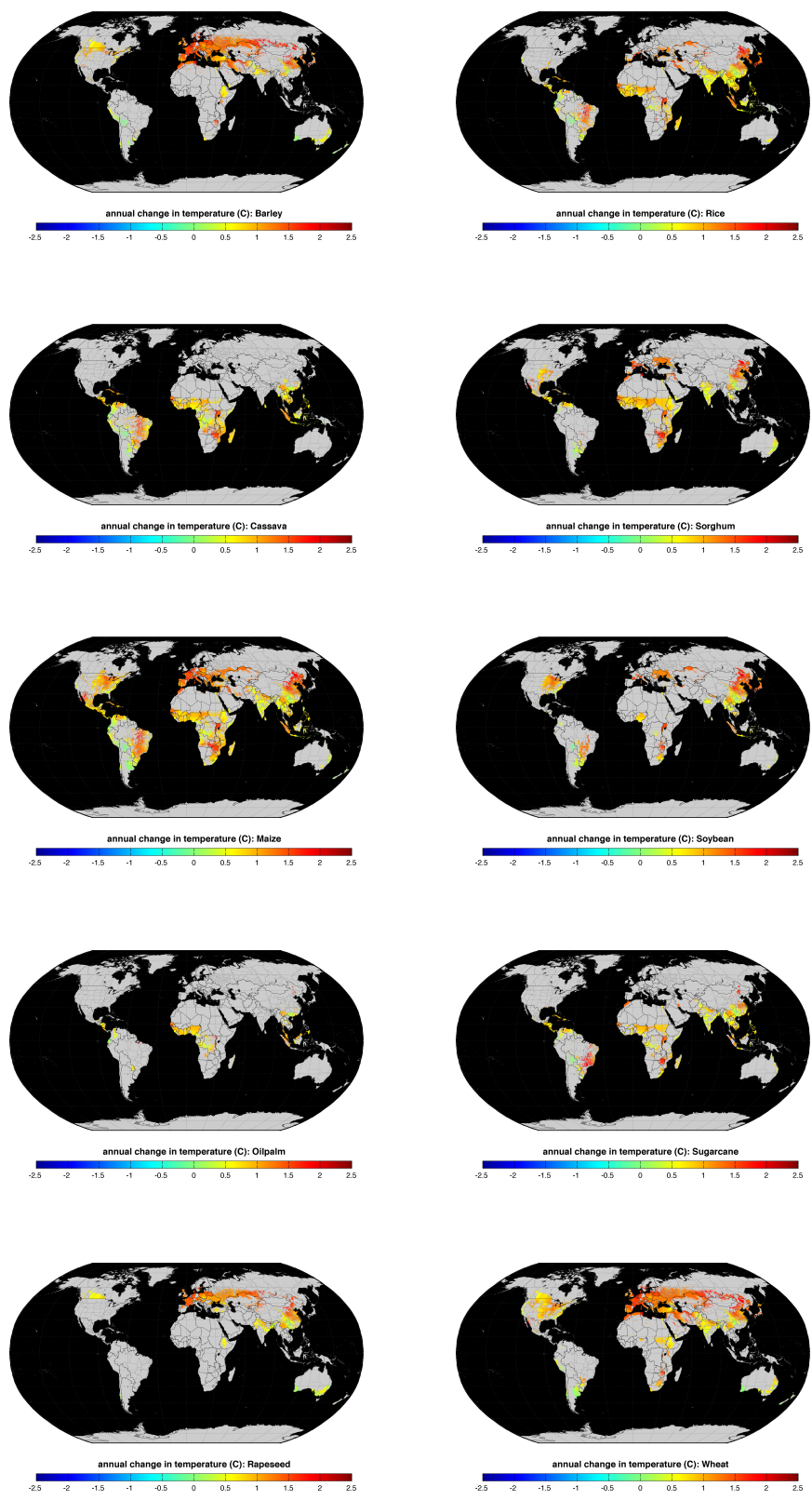

Supplement: S2 Fig — (PDF) [file pone.0217148.s003.pdf]

S3 Fig Seasonal monthly climatological precipitation change map for the ten crops by PU.

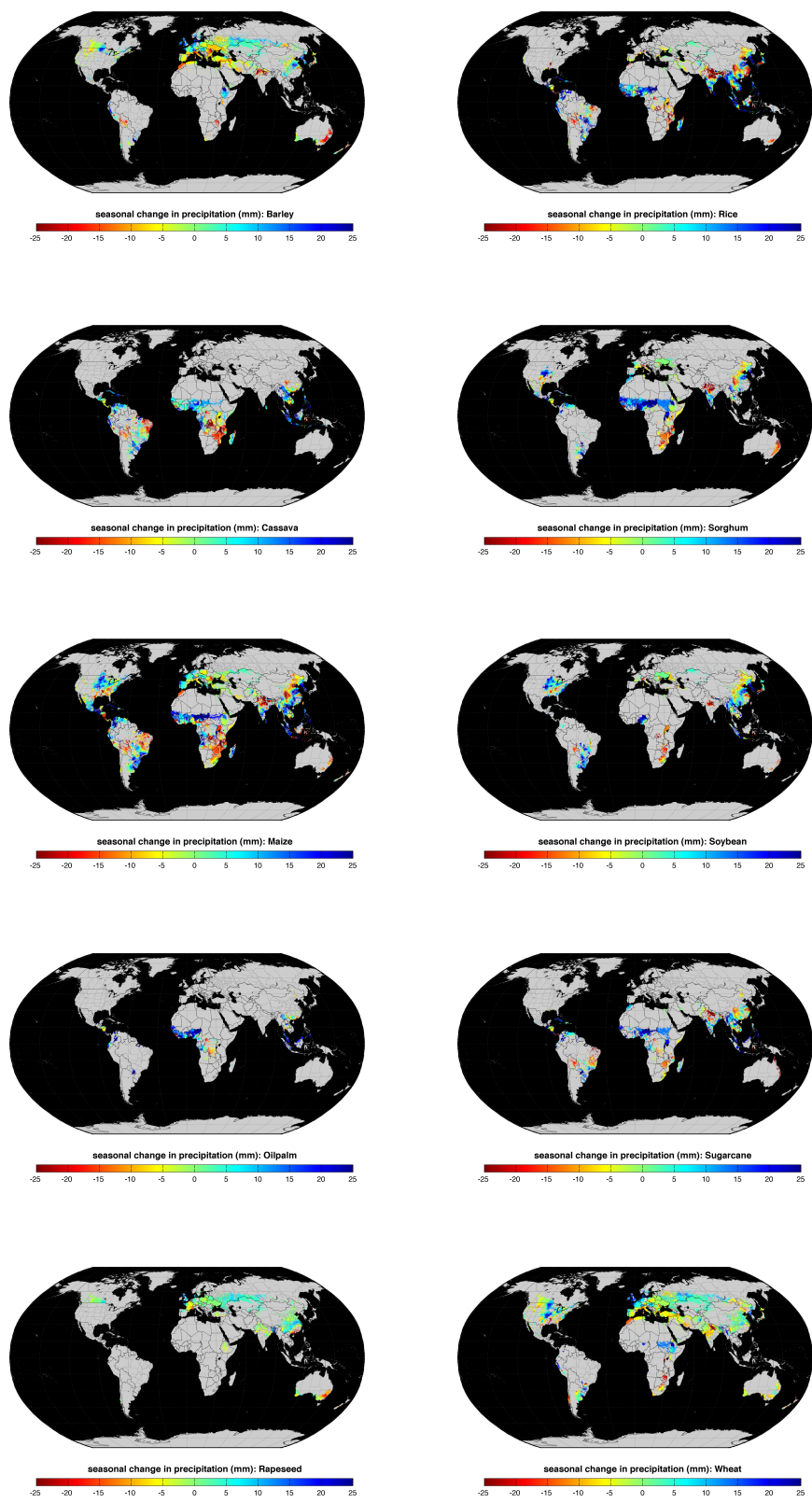

Supplement: S3 Fig — (PDF) [file pone.0217148.s004.pdf]

S4 Fig Annual monthly climatological precipitation change map for the ten crops by PU.

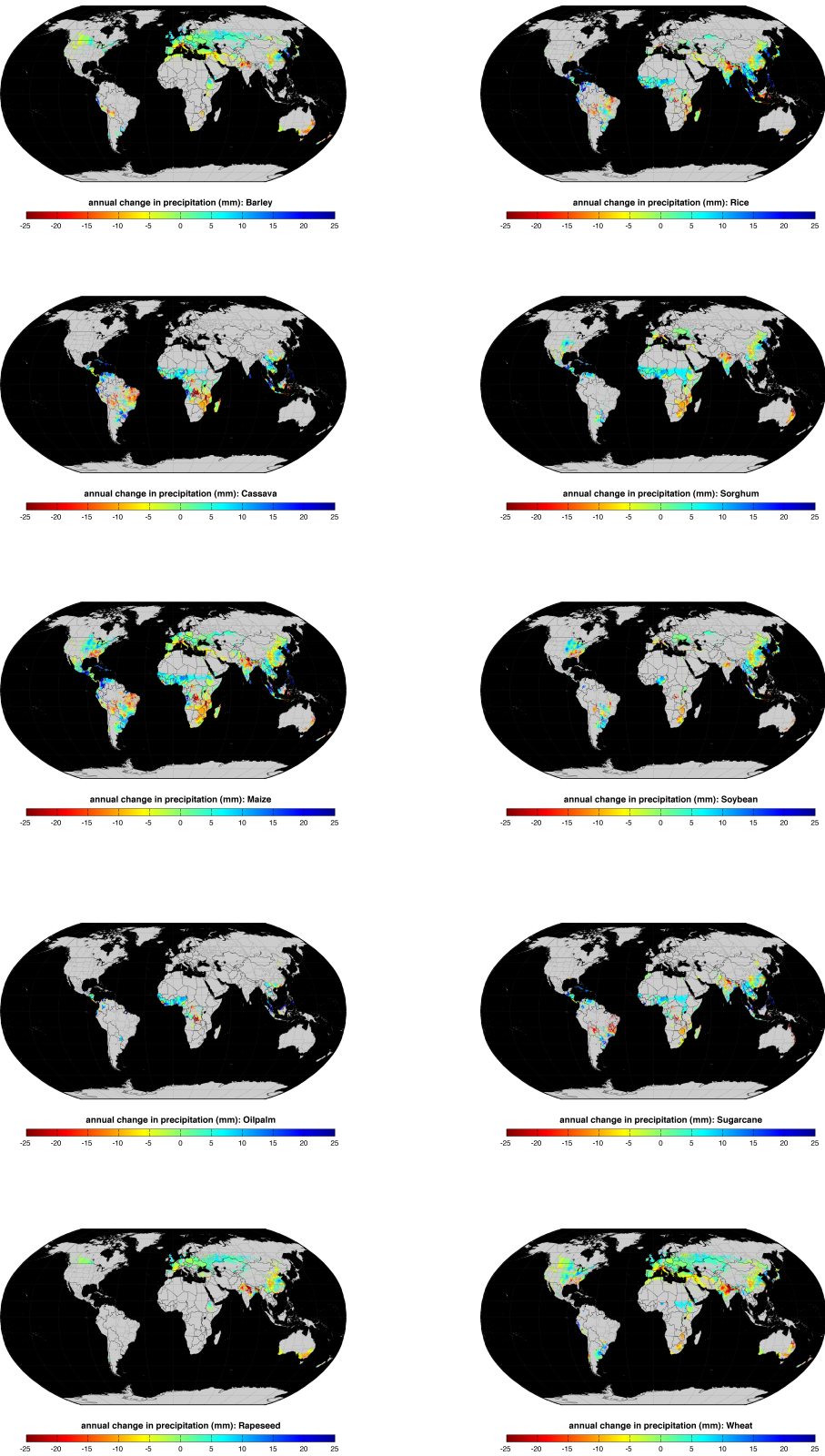

Supplement: S4 Fig — (PDF) [file pone.0217148.s005.pdf]

S8 Fig Coefficient of determination of the model for the ten crops mapped by PU.

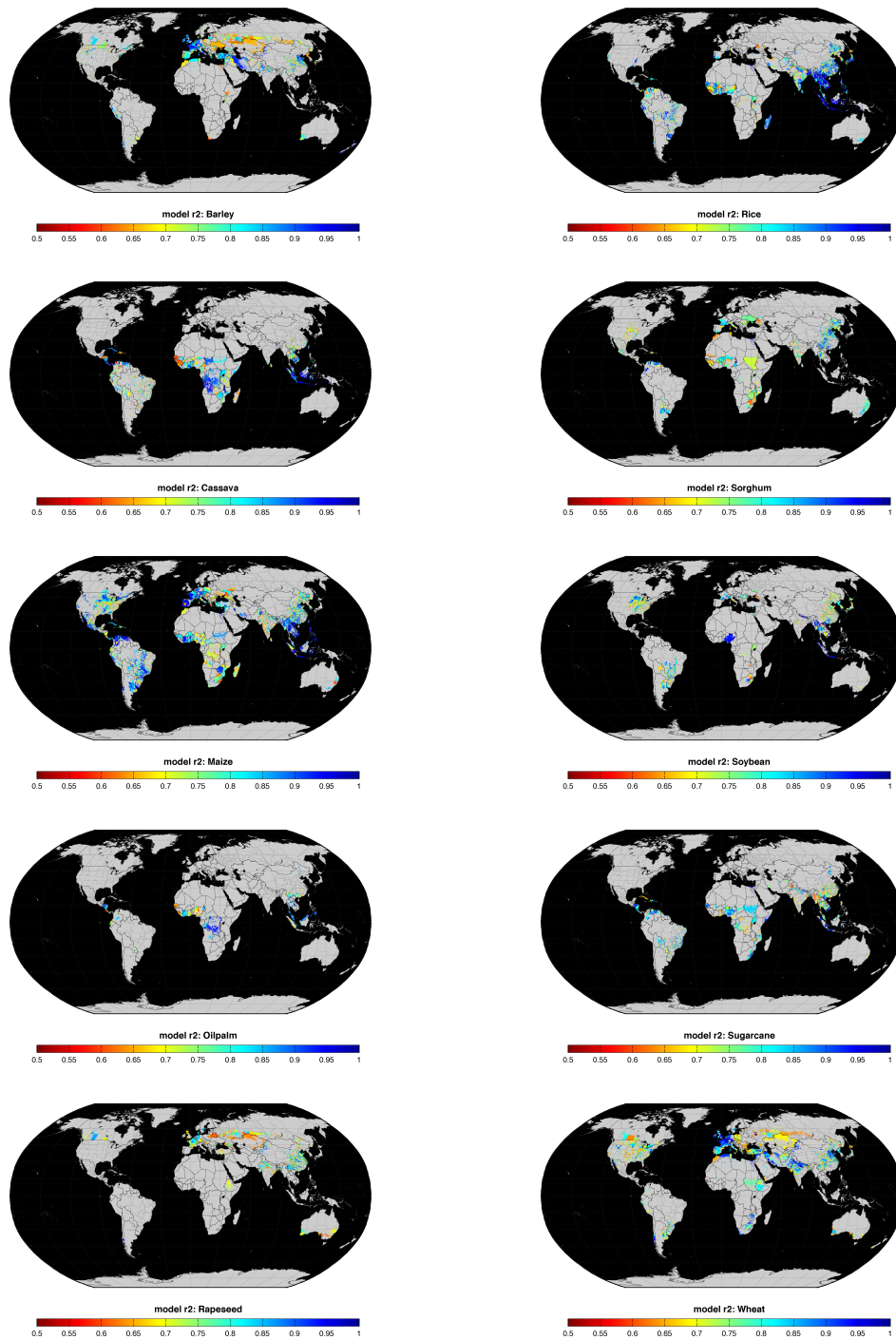

Supplement: S8 Fig — (PDF) [file pone.0217148.s009.pdf]
