# Supplementary material for: Climate change has likely already affected global food production
Source: PLoS One. 2019 May 31;14(5):e0217148. doi: 10.1371/journal.pone.0217148 (PMC6544233; doi:10.1371/journal.pone.0217148)

S5 Fig Three examples (a), (b), & (c) when model cannot be used (plotted in the maps of Fig 1 in the main text as white colored areas).

Maize, Cameron county, Texas, USA. Model p value: 0.67. Model R^2: 0.35, MSE: 1.44 tons/ha. If model was used for prediction then climate trend impact would be: -0.7 tons/ha/year

a

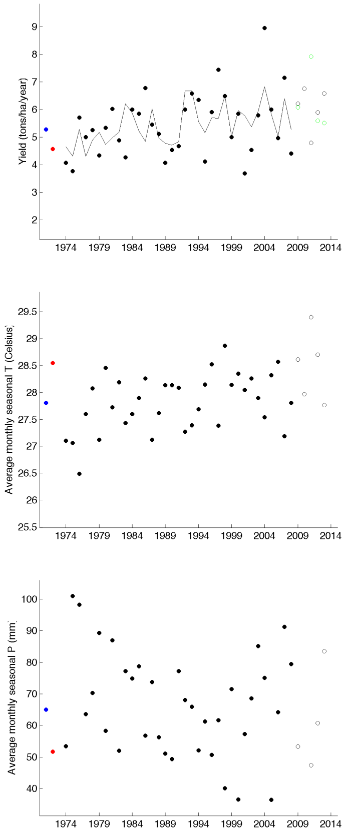

Rice, Gaza province, Mozambique. Model p value: 0.56. Model R^2: 0.39, MSE: 0.14 tons/ha. Model cannot be used for prediction as yield for current climate is: -0.18 tons/ha/year

b

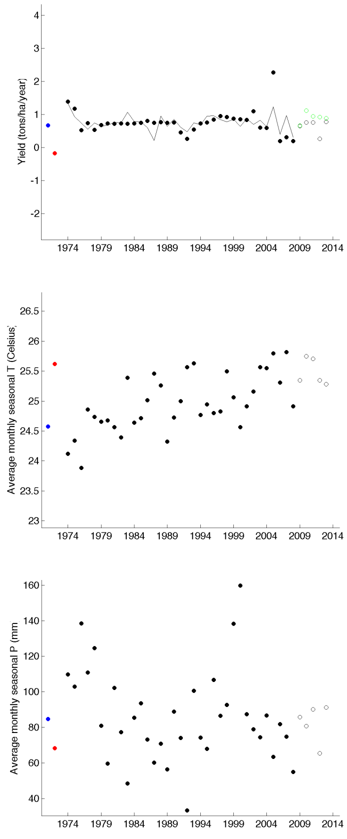

Wheat, Languedoc Rousillon, Mediterranean, France. Model p value: 0.32. Model R^2: 0.46, MSE: 0.49 tons/ha. If model was used for prediction then climate trend impact would be: -1.54 tons/ha/year

c

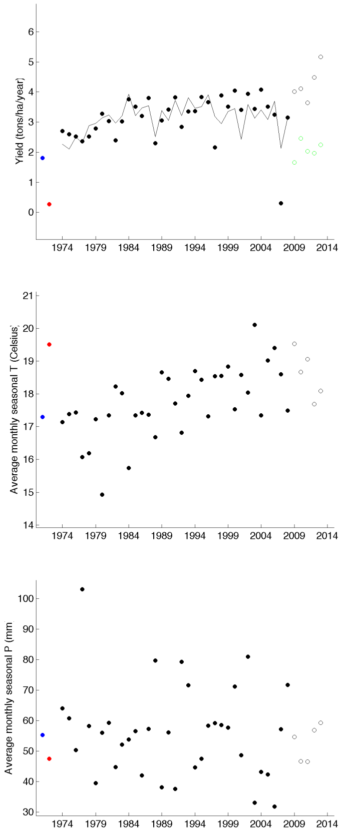

Supplement: S5 Fig — Three examples (a), (b), & (c) when model cannot be used (plotted in the maps of Fig 1 in the main text as white colored areas). (PDF) [file pone.0217148.s006.pdf]
