# Supplementary material for: Climate change has likely already affected global food production
Source: PLoS One. 2019 May 31;14(5):e0217148. doi: 10.1371/journal.pone.0217148 (PMC6544233; doi:10.1371/journal.pone.0217148)
Supplement: S6 Fig — The models were then used for historical (open blue circles) and current (open red circles) climate conditions. Out-of-sample predictions do not occur as the historical and current conditions are bounded within the training weather conditions. (a) Maize (USA), (b) Rice (China), (c) Wheat (India), (d) Soybean (USA) (e) Maize (Mexico) and (f) Maize (China). Training weather data size is (N) X (35) and shown figures are for seasonal monthly conditions. Annual monthly conditions showed similar results (not shown). (PDF) [file pone.0217148.s007.pdf]

S6 Fig Weather information used for constructing the regression model for example crop and country cases (solid black filled circles). The models were then used for historical (open blue circles) and current (open red circles) climate conditions. Out-of-sample predictions do not occur as the historical and current conditions are bounded within the training weather conditions. (a) Maize (USA), (b) Rice (China), (c) Wheat (India), (d) Soybean (USA) (e) Maize (Mexico) and (f) Maize (China). Training weather data size is (N) X (35) and shown figures are for seasonal monthly conditions. Annual monthly conditions showed similar results (not shown).

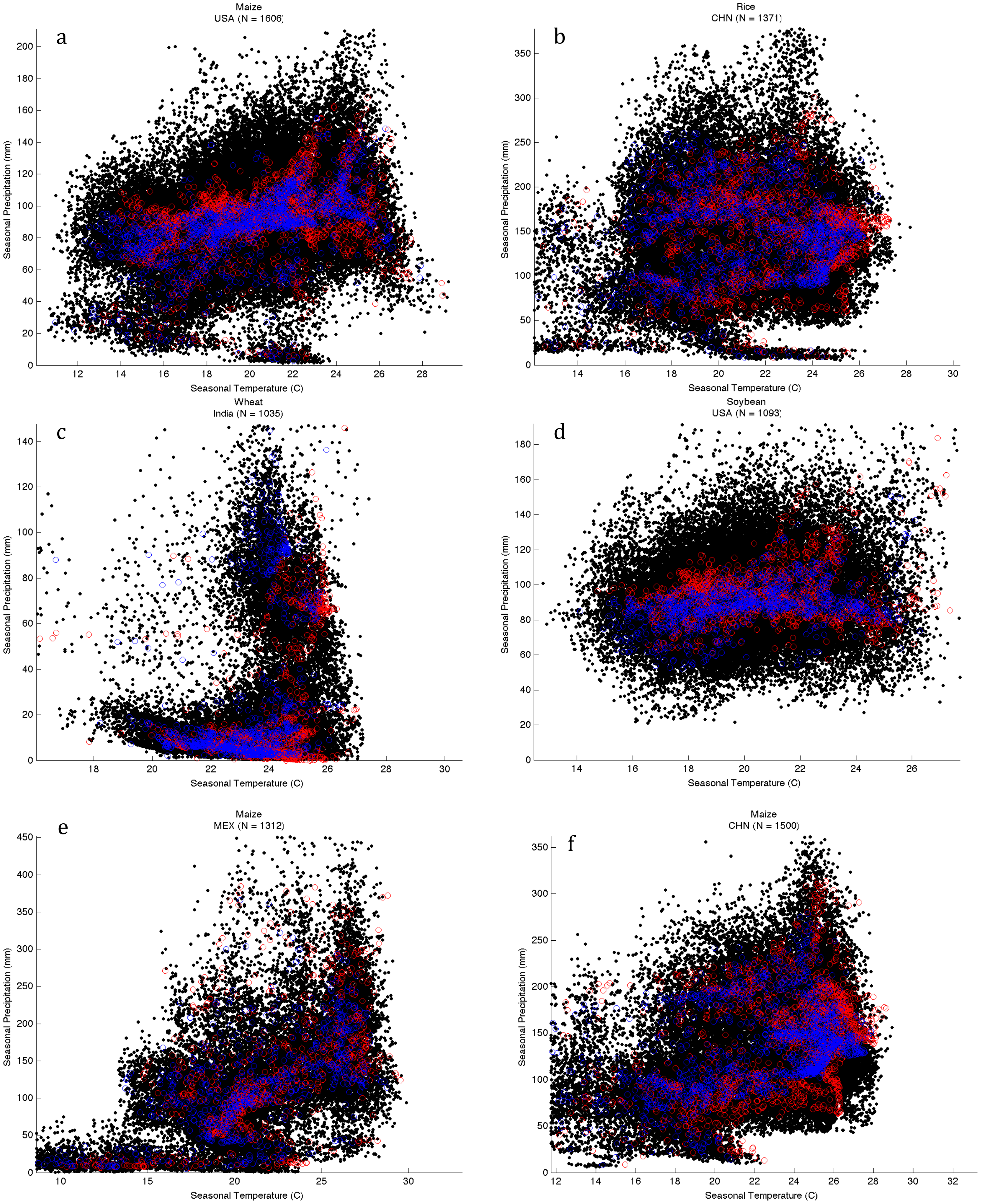

Maize
USA (N = 1606)
a
Rice
CHN (N = 1371)
b
Wheat
India (N = 1035)
c
Soybean
USA (N = 1093)
d
Maize
MEX (N = 1312)
e
Maize
CHN (N = 1500)
f
Seasonal Precipitation (mm)
Seasonal Temperature (C)
