# Supplementary material for: Climate change has likely already affected global food production
Source: PLoS One. 2019 May 31;14(5):e0217148. doi: 10.1371/journal.pone.0217148 (PMC6544233; doi:10.1371/journal.pone.0217148)

S7 Fig Areas with statistically significant white noise error (at p = 0.01 level, red colored regions, 0% to 8% of all studied regions depending on the crop) as determined from Ljung-Box Q-Tests for autocorrelation in the residuals per crop and political unit.

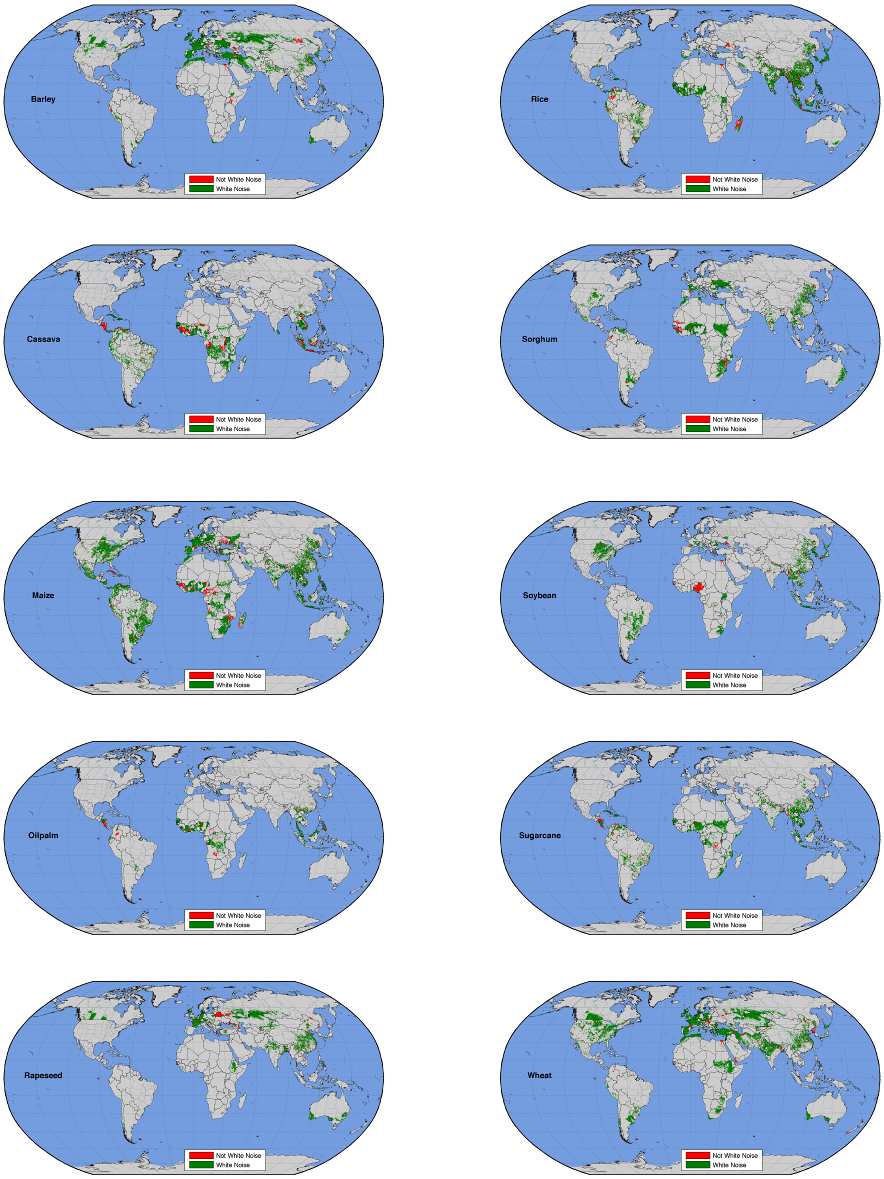

Supplement: S7 Fig — (PDF) [file pone.0217148.s008.pdf]
