# Supplementary material for: Climate change has likely already affected global food production
Source: PLoS One. 2019 May 31;14(5):e0217148. doi: 10.1371/journal.pone.0217148 (PMC6544233; doi:10.1371/journal.pone.0217148)

S9 Fig Change in crop yield due to only temperature climatological change (holding the precipitation variables at historical levels).

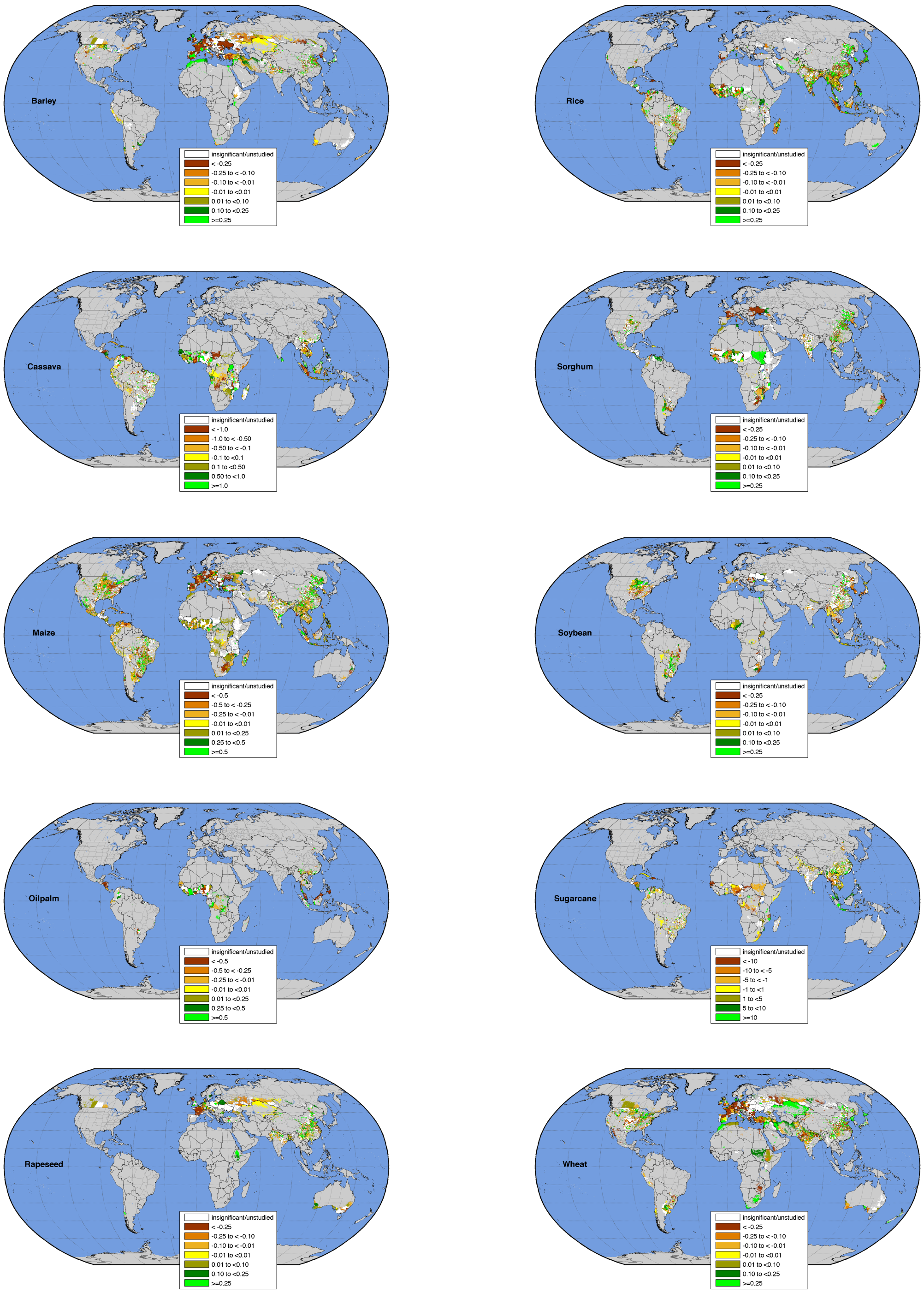

Supplement: S9 Fig — (PDF) [file pone.0217148.s010.pdf]
