# Supplementary material for: Climate change has likely already affected global food production
Source: PLoS One. 2019 May 31;14(5):e0217148. doi: 10.1371/journal.pone.0217148 (PMC6544233; doi:10.1371/journal.pone.0217148)

S10 Fig Change in crop yield due to only precipitation climatological change (holding the temperature variables at historical levels).

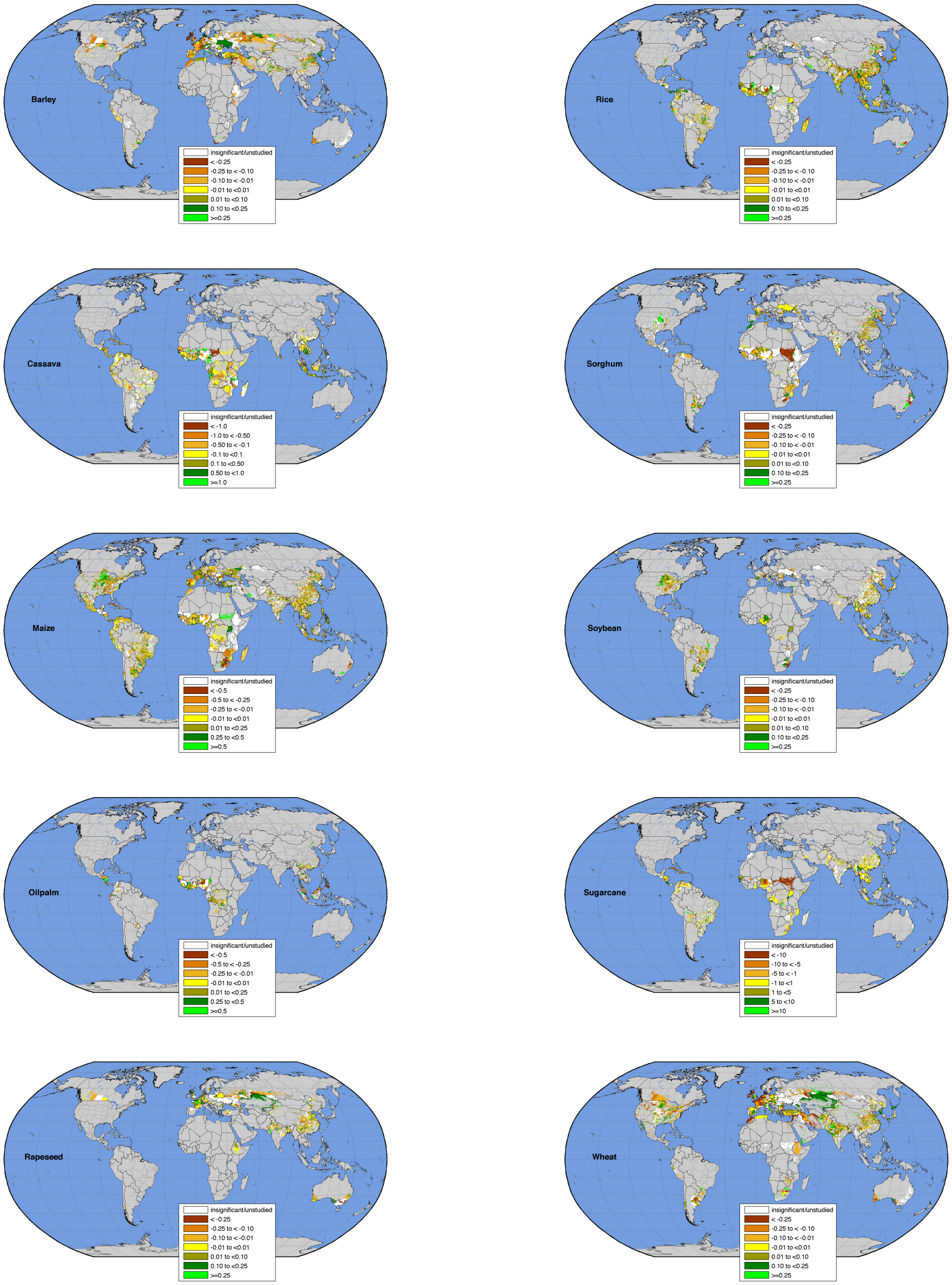

Supplement: S10 Fig — (PDF) [file pone.0217148.s011.pdf]
