# Supplementary material for: Climate change has likely already affected global food production
Source: PLoS One. 2019 May 31;14(5):e0217148. doi: 10.1371/journal.pone.0217148 (PMC6544233; doi:10.1371/journal.pone.0217148)

S11 Fig Maps of model Mean Squared Errors (MSE) (tons/ha/year). (Note the variable legends which goes from zero to about half of global averaged yields – Table S4).

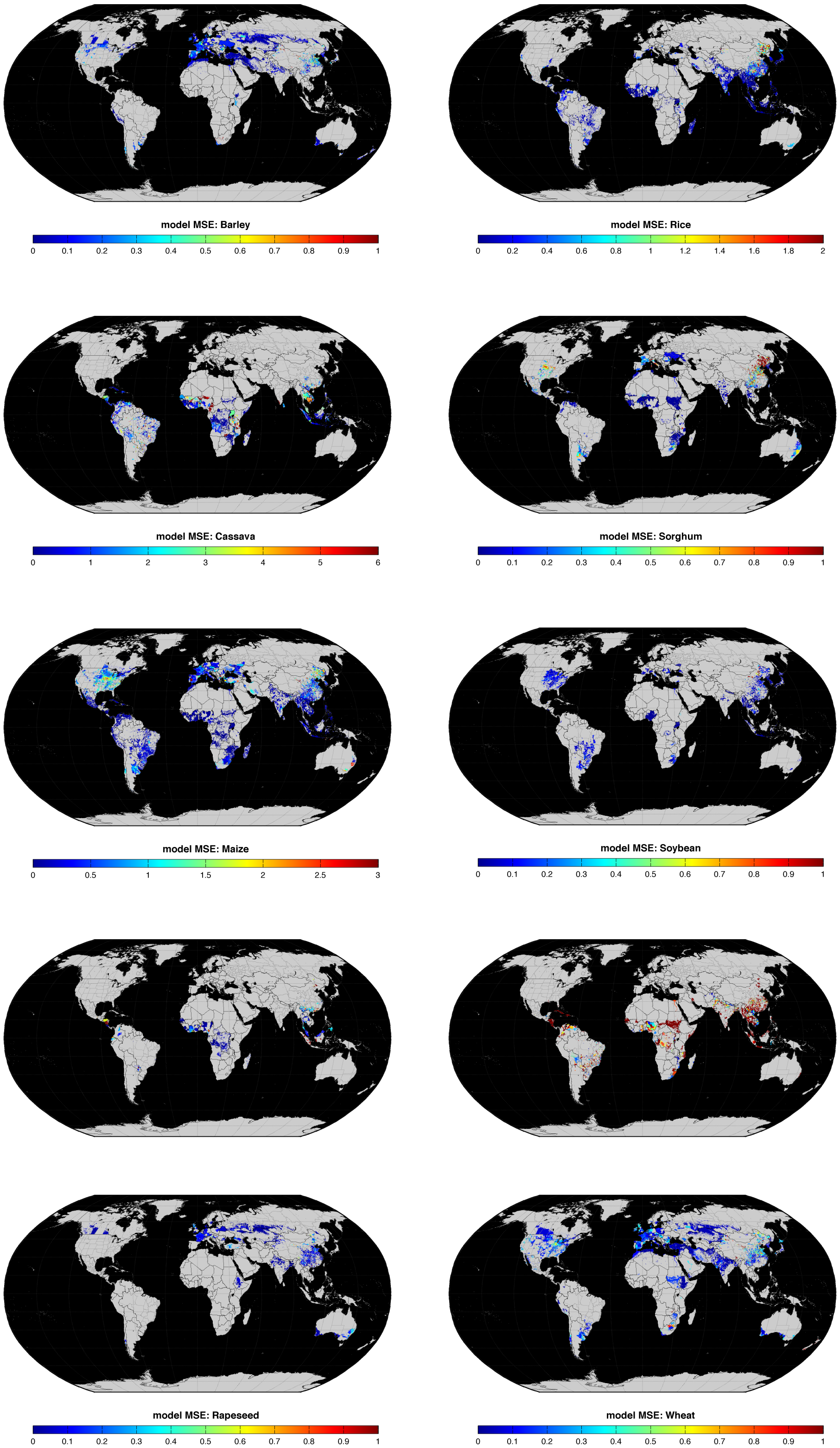

Supplement: S11 Fig — (Note the variable legends which goes from zero to about half of global averaged yields–S4 Table). (PDF) [file pone.0217148.s012.pdf]
