# Supplementary material for: Climate change has likely already affected global food production
Source: PLoS One. 2019 May 31;14(5):e0217148. doi: 10.1371/journal.pone.0217148 (PMC6544233; doi:10.1371/journal.pone.0217148)

S12 Fig Sensitivity maps to seasonal P, T, $P^2$ and $T^2$ for maize, rice and wheat where sensitivity = coefficient / change.

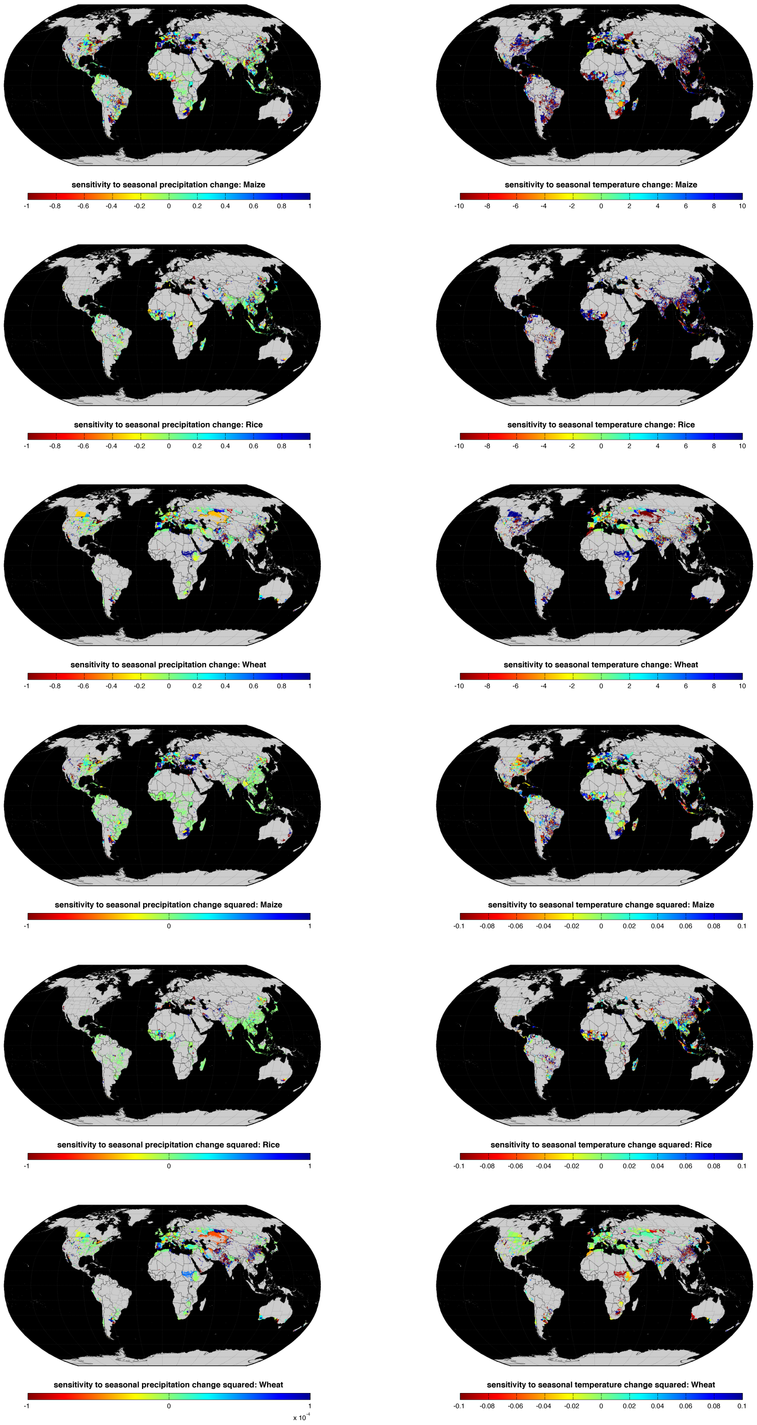

Supplement: S12 Fig — (PDF) [file pone.0217148.s013.pdf]
