# Supplementary material for: Climate change has likely already affected global food production
Source: PLoS One. 2019 May 31;14(5):e0217148. doi: 10.1371/journal.pone.0217148 (PMC6544233; doi:10.1371/journal.pone.0217148)
Supplement: S1 Table — Change is the difference between monthly average current and historical value. (PDF) [file pone.0217148.s014.pdf]

S1 Table Climatological change in temperature and precipitation (values are averaged over all harvested croplands including those with insignificant climate change impact or unstudied due to data limitations). Change is the difference between monthly average current and historical value.

	Crop	Temperature change (ºC)	Precipitation change (mm)
Seasonal changes			
1	Barley	+ 1.2	- 1.7
2	Cassava	+ 0.8	+ 3.1
3	Maize	+ 0.8	+ 3.0
4	Oilpalm	+ 0.8	+ 19.2
5	Rapeseed	+ 1.0	+ 0.6
6	Rice	+ 0.7	- 0.3
7	Sorghum	+ 0.7	+ 9.3
8	Soybean	+ 0.7	+ 5.6
9	Sugarcane	+ 0.5	- 3.4
10	Wheat	+ 0.9	- 0.7
Annual changes			
1	Barley	+ 1.2	- 0.1
2	Cassava	+ 0.8	+ 1.8
3	Maize	+ 1.0	+ 1.4
4	Oilpalm	+ 0.9	+ 14.7
5	Rapeseed	+ 0.8	- 2.6
6	Rice	+ 0.7	- 0.2
7	Sorghum	+ 0.8	+ 3.0
8	Soybean	+ 0.9	+ 1.6
9	Sugarcane	+ 0.6	- 2.7
10	Wheat	+ 1.0	- 1.6
Harvested hectares averaged over (million ha (Mha))			
1	Barley	51.5	
2	Cassava	16.9	
3	Maize	133.2	
4	Oilpalm	10.8	
5	Rapeseed	23.9	
6	Rice	141.8	
7	Sorghum	40.1	
8	Soybean	74.3	
9	Sugarcane	17.0	
10	Wheat	196.8	
