# Supplementary material for: Climate change has likely already affected global food production
Source: PLoS One. 2019 May 31;14(5):e0217148. doi: 10.1371/journal.pone.0217148 (PMC6544233; doi:10.1371/journal.pone.0217148)
Supplement: S2 Table — (PDF) [file pone.0217148.s015.pdf]

S2 Table Harvested area weighted average model yield prediction errors for 2009 to 2013 globally (percentage off from observations).

Barley	-2.04
Cassava	-3.05
Maize	1.09
Oil Palm	2.51
Rapeseed	-6.23
Rice	-1.90
Sorghum	2.99
Soybean	4.00
Sugarcane	-0.04
Wheat	-3.00
