# Supplementary material for: Climate change has likely already affected global food production
Source: PLoS One. 2019 May 31;14(5):e0217148. doi: 10.1371/journal.pone.0217148 (PMC6544233; doi:10.1371/journal.pone.0217148)
Supplement: S3 Table — (PDF) [file pone.0217148.s016.pdf]

S3 Table Production weighted coefficient of determination averaged globally per crop

Barley	0.789
Maize	0.821
Cassava	0.827
Oil Palm	0.837
Rapeseed	0.788
Rice	0.868
Sorghum	0.758
Soybean	0.776
Sugarcane	0.793
Wheat	0.818
