# Supplementary material for: Climate change has likely already affected global food production
Source: PLoS One. 2019 May 31;14(5):e0217148. doi: 10.1371/journal.pone.0217148 (PMC6544233; doi:10.1371/journal.pone.0217148)
Supplement: S5 Table — Computed changes are not additive). (PDF) [file pone.0217148.s018.pdf]

S5 Table Percentage yield changed w.r.t historical global scale yield. (Yields under current T or P change are only over areas where change was detected and historical yields are over all cropped areas. Computed changes are not additive).

	Temperature change impact (%)	Precipitation change impact (%)
Barley	-9.75	-2.05
Cassava	-0.10	0.22
Maize	-0.30	1.71
Oilpalm	-15.92	-0.17
Rapeseed	4.23	1.24
Rice	0.12	-0.03
Sorghum	8.81	-8.73
Soybean	4.04	1.20
Sugarcane	2.67	-0.43
Wheat	0.25	-0.74
