# Supplementary material for: Climate change has likely already affected global food production
Source: PLoS One. 2019 May 31;14(5):e0217148. doi: 10.1371/journal.pone.0217148 (PMC6544233; doi:10.1371/journal.pone.0217148)
Supplement: S6 Table — (PDF) [file pone.0217148.s019.pdf]

S6 Table Globally averaged Mean Square Error (MSE) (tons/ha) averaged over the harvested areas studied. Current yield refers to averaged yields over areas reported over years 2004-2008.

	MSE	Current Yield
Barley	0.11	2.59
Cassava	1.72	12.15
Maize	0.58	5.84
Oil Palm	2.02	15.40
Rapeseed	0.07	1.85
Rice	0.20	4.36
Sorghum	0.09	1.49
Soybean	0.11	2.57
Sugarcane	41.01	65.71
Wheat	0.13	3.05
